# Outer-Sphere CO Release Mechanism in the Methanol-to-Syngas Reaction Catalyzed by a Ru-PNP Pincer Complex

**DOI:** 10.1021/acscatal.4c06818

**Published:** 2025-03-12

**Authors:** Jiali Liu, Raquel J. Rama, Tomás Cordero-Lanzac, Mohamed E. A. Safy, Robert Franke, Ainara Nova

**Affiliations:** a Evonik Oxeno GmbH & Co. KG, Paul-Baumann-Str. 1, Marl 45772, Germany; b Lehrstuhl für Theoretische Chemie, Ruhr-Universität Bochum, Bochum 44780, Germany; c Center for Materials Science and Nanotechnology (SMN), Department of Chemistry, University of Oslo, Oslo 0315, Norway; d Hylleraas Centre for Quantum Molecular Sciences, Department of Chemistry, University of Oslo, Oslo N-0315, Norway

**Keywords:** ruthenium, pincer complexes, DFT calculations, microkinetic modeling, syngas release, reaction mechanism, bifunctional catalyst

## Abstract

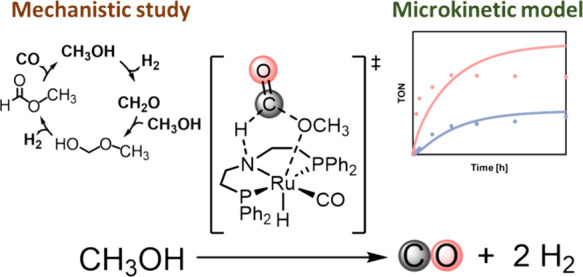

Methanol can be used as a surrogate molecule for CO and H_2_ in the synthesis of a large variety of chemicals. In this work, the mechanism for the methanol-to-syngas reaction catalyzed by a Ru-PNP complex was studied using density functional theory. In the proposed mechanism, the CO is directly released from the methyl formate intermediate, forming a Ru-OCH_3_ species. The preference for this pathway compared to others proposed in literature was supported by a microkinetic model constructed from the computed Gibbs free energies and coupled to a liquid–vapor batch reactor describing the gas phase composition. After including energy corrections of ≤6 kcal mol^–1^ to three organic intermediates and CO, our model could reproduce the experimental CO and H_2_ turnover numbers over the time previously reported. Further, this model was used to evaluate the influence of solvent polarity and methanol concentration on the formation of products and catalyst resting states. These results suggest that in methanol, CO formation is limited by the organic reaction thermodynamics, whereas in toluene, it is limited by Ru–CO formation. Overall, this work shows the potential of microkinetic models to benchmark reaction mechanisms and computational methods and provide the relevant information required for catalyst design.

## Introduction

Methanol, obtained from captured CO_2_ and green H_2_, has the possibility to replace fossil fuels for energy storage, ground transportation, and raw materials for synthetic hydrocarbons.^1,2^ This route has high industrial potential due to the required reaction conditions and the relevance of methanol as a platform chemical. Methanol is a versatile organic solvent and is used as a feedstock in the production of fine and bulk chemicals including polymers. As an example, methanol can be used as a surrogate molecule for H_2_ and/or CO to synthesize a large variety of products containing alcohol, aldehyde, amine, amide, and sulfonamide functional groups.^3−5^

The mechanism for the decomposition of methanol into H_2_ and CO (syngas) catalyzed by metallic surfaces has been thoroughly investigated by experiments and computational methods.^6−12^ This transformation has been less studied on metal oxide and zeolite heterogeneous catalysts,^13,14^ even though the CO formation from methanol plays a critical role in the conversion of methanol to hydrocarbons.^15,16^ Recently, the mechanistic similarity between heterogeneous and homogeneous catalysts in hydrogen transfer reactions has been reported, with particular interest in catalysts with Lewis acid–base pairs such as metal oxides, M-N bifunctional homogeneous catalysts, and frustrated Lewis pairs.^17,18^ This is a significant advance since the isolated active site in homogeneous catalysts facilitates studying their mechanism of action.

**Figure 1:**
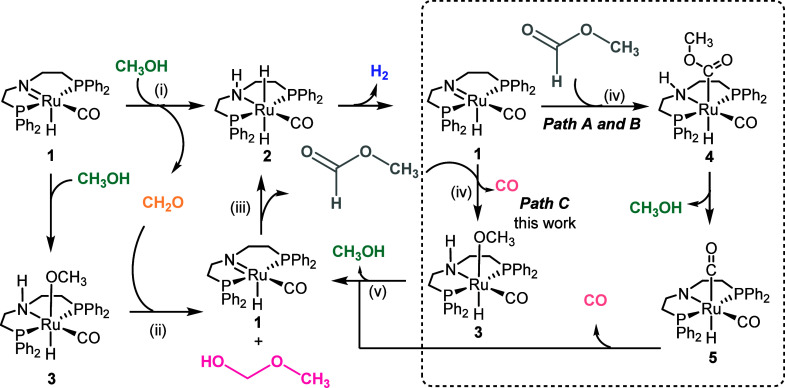
Reaction steps proposed for the methanol-to-syngas reaction catalyzed by the Ru complex **1**: (i) methanol dehydrogenation, (ii) methanol and formaldehyde coupling, (iii) methoxymethanol dehydrogenation, (iv) methyl formate decarbonylation, and (v) catalyst recovery.

In 2021, the formation of syngas from methanol was achieved by Leitner’s group using RuH(CO)(BH_4_)(HN(C_2_H_4_PPh_2_)_2_) and MnBr(CO)_2_(HN(C_2_H_4_P*i*Pr_2_)_2_) complexes, yielding a ratio of CO:H_2_ close to the stoichiometric 1:2.^19^ In this work, two catalytic cycles were proposed: one for the decarbonylation of formaldehyde resulting from methanol dehydrogenation and the other for the decarbonylation of methyl formate, which is produced by the reaction of formaldehyde with methanol. The computational study of this reaction has recently appeared in two different publications.^20,21^ In both studies, it was proposed that the reaction does not proceed by the decarbonylation of formaldehyde but proceeds only via methyl formate. The mechanism for the overall reaction consists of the following steps: (i) methanol dehydrogenation, (ii) methanol and formaldehyde coupling, (iii) methoxymethanol dehydrogenation, and (iv) methyl formate decarbonylation (Figure 1). While the mechanism for many of these steps is similar in the two studies and has been proposed for other reactions, such as the aqueous methanol-reforming,^22,23^ the mechanism for the methyl formate decarbonylation step has some differences. In particular on the orientation of the CH_3_OC=O fragment, resulting from the deprotonation of methyl formate by the Ru-nitrogen complex, which coordinates Ru by either the C=O group (path A) or the OCH_3_ group (path B). Despite this difference, both reactions yield the same Ru–COOCH_3_ intermediate (**4**). The energy barriers for both routes (A and B) were similar; however, they were obtained using different computational methods. In addition, the computed energies were not correlated with the experimental formation of H_2_ and CO obtained by Leitner and co-workers, and they were difficult to compare because several Ru intermediates participate in more than one catalytic cycle (Figure 1).^19^ Especially in these cases, microkinetic models fed with computed energies are useful, offering the possibility to simulate the formation of intermediates and products over time, which can be directly compared with experimental data.^24−26^

In this work, density functional theory (DFT) methods were used to compute the complete reaction mechanism for the methanol-to-syngas reaction using methanol and toluene solvents. In distinction to previous investigations, these data were used to construct microkinetic models for distinct mechanisms (paths A and C in Figure 1), which were coupled with a liquid–vapor batch reactor model designed from the reported experimental setup. After an energy fitting of the organic reaction thermodynamics, only path C showed good agreement with the experimental H_2_ and CO formation. In this mechanism, which resembles the one proposed for decarbonylation reactions catalyzed by an organic base,^27^ the methyl formate decarbonylation yields the Ru-methoxy intermediate **3** by direct CO release. This reaction is preferred in toluene compared to methanol due to the lower polarity of the organic products and the lower stability of the Ru-alkoxy intermediates.

## Results and Discussion

### Decarbonylation of Methyl Formate

**Figure 2:**
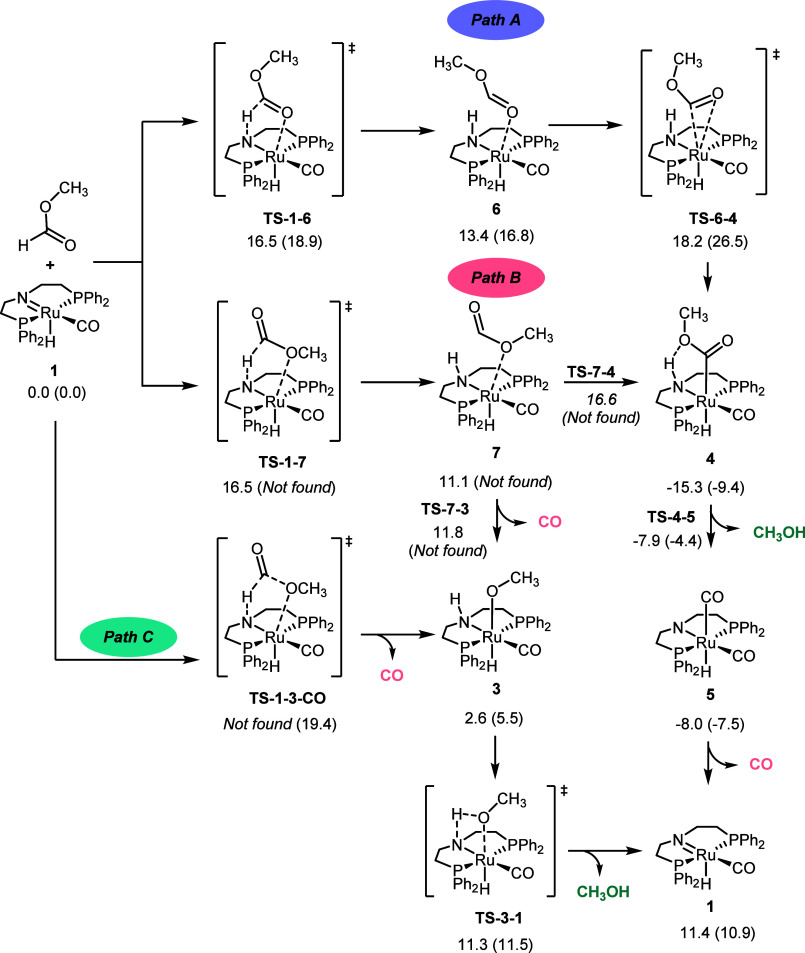
Gibbs Free energies (in kcal mol^–1^) in methanol, and toluene in parentheses, for the decarbonylation of methyl formate via paths A, B, and C.

The Gibbs Free energies obtained for the decarbonylation reaction of methyl formate catalyzed by **1** in methanol and toluene as the solvent are shown in Figure 2. These solvents were selected because they were used in the experimental study of the methanol-to-syngas reaction.^19^ In addition, path A was previously studied in toluene.^20^ Therefore, we were also interested in analyzing the influence of solvent in the reaction by comparing results in methanol, toluene, and toluene/methanol (see Figures S1–S4 and Tables S1–S4 in the Supporting Information).

The two pathways previously proposed in the literature, A and B, start with deprotonation of methyl formate by the amido ligand in **1** (**TS-1-6** and **TS-1-7**), followed by ligand rearrangement (**TS-6-4** and **TS-7-4**), yielding intermediate **4**. In methanol, the transition state with the highest energy in path A is **TS-6-4**, where the CH_3_OC=OCH_3_ ligand changes its coordination from k^1^-O to σ^1^-C, with an energy of 18.2 kcal mol^–1^. In path B, the analogous TS (**TS-7-4**) was found at 16.6 kcal mol^–1^ and has a similar energy to that of the deprotonation step via **TS-1-7** (16.5 kcal mol^–1^). In both pathways, the formation of compound **4** is highly exergonic by 15.3 kcal mol^–1^. Methanol release from this intermediate has an energy barrier of 7.4 kcal mol^–1^ (**TS-4-5**) and yields a dicarbonyl Ru intermediate (**5**), which has been experimentally detected.^19^ This reaction and the CO ligand dissociation to recover catalyst **1** are both endergonic, with energies of 7.3 and 19.4 kcal mol^–1^, respectively. Together, they yield an energy of 26.7 kcal mol^–1^ for the overall process, suggesting that the methanol and CO release from intermediate **4** is one of the limiting steps.

Calculations of path A and B in toluene gave significantly different results. All intermediates and transition states increased in energy; in particular, **TS-6-4** in path A went from 18.2 to 26.5 kcal mol^–1^ in toluene. In addition, intermediate **7** in path B could not be located as a minimum; instead, it was found to be a transition state yielding the concerted deprotonation of methyl formate and C-OCH_3_ bond cleavage (**TS-1-3-CO**, path C). This step, with an energy barrier of 19.4 kcal mol^–1^, yields the methoxy intermediate **3**, which is involved in other steps of the methanol-to-syngas reaction (Figure 1). Energetically, path C is not only lower than A by 7.1 kcal mol^–1^ but it also prevents the formation of **4**, which is highly unfavorable for the reaction. In this case, the methanol release from **3** has an energy barrier of 6.0 kcal mol^–1^ and is endergonic by a similar energy. In addition, the formation of **5**, experimentally observed, is still possible by the addition of the released CO to the Ru complex **1** (see the Microkinetic Modeling section).^19^

The concerted decarbonylation reaction in toluene made us consider path C also in methanol. In this case, instead of being concerted, the CO elimination starts with the deprotonation of methyl formate via **TS-1-7** (as in path B) followed by the C-OCH_3_ bond cleavage (**TS-7-3**). From these two steps, deprotonation has the highest energy barrier (16.5 kcal mol^–1^), and the CO release from intermediate **7** has an energy cost lower than 1 kcal mol^–1^. Comparing this result with that of **TS-7-4** in path B, which is 5.5 kcal mol^–1^ higher than **7**, indicates that path C is also preferred in methanol.

### Decarbonylation of Formaldehyde

**Figure 3:**
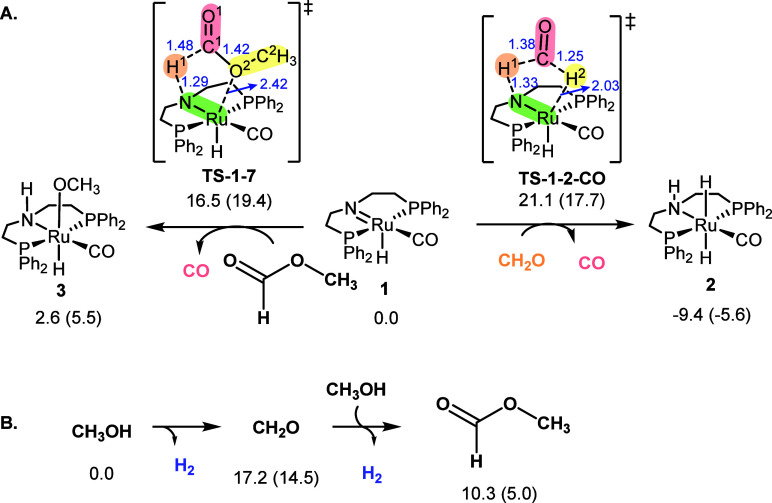
Gibbs Free energies (in kcal mol^–1^) in methanol and toluene in parentheses for the decarbonylation of methyl formate and formaldehyde via path C (A) and formation of these species from methanol (B). Blue numbers are optimized bond lengths in a methanol solvent.

The decarbonylation reaction following path C can be understood as the fragmentation of methyl formate into H^+^, CO, and CH_3_O^–^ by the N–Ru base-acid pair. A similar fragmentation could occur with formaldehyde, in this case to H^+^, CO, and H^–^ (Figure 3A). In methanol, the decarbonylation of formaldehyde by path C follows a concerted mechanism with a higher energy barrier than that computed with methyl formate (21.1 kcal mol^–1^ for formaldehyde and 16.5 kcal mol^–1^ for methyl formate). In toluene, the reaction also follows a concerted mechanism, with formaldehyde having the lowest energy barrier (17.7 kcal mol^–1^) compared with that of methyl formate (19.4 kcal mol^–1^).

An analysis of the changes in the atomic charges of the H^1^, CO, H^2^/OCH_3_, and Ru–N fragments from reactants to the transition state (see Table 1 and Tables S5 and S6) shows that electrons are transferred from H^1^ to Ru–N in the case of formaldehyde and to the CO fragment in methyl formate. One of the reasons for this difference is the more significant negative charge of CO in formaldehyde (−0.244 e) compared to methyl formate (0.061 e), in which the carbonyl group is more oxidized. It is probably the increase in the negative charge of CO in the case of methyl formate, which increases the polarity of the transition state, that makes the decarbonylation reaction preferred in methanol. In contrast, **TS-1–2-CO** is preferred in the less polar toluene solvent since the Ru–N fragment is less exposed to the solvent. For both substrates, H^2^/OCH_3_ is slightly reduced.

**Table 1:** NPA Analysis of Key Atoms in Structures Optimized in a Methanol Solvent

fragments	Δ*q* (**1+CH**_**2**_**O → TS-**1–2**-CO**)	Δ*q* (**1+ HCOOCH**_**3**_**→ TS-**1–7)
H^1^	0.175	0.177
CO	–0.094	–0.244
H^2^ or OCH_3_	–0.050	–0.044
RuN	–0.194	–0.006

When considering the decarbonylation energy barriers from both methyl formate and formaldehyde from **1**, they both seem reasonable and feasible under the reaction conditions. However, the needed Gibbs free energy to form these intermediates from methanol (Δ*G* in Figure 3B) should be added to the decarbonylation energy barriers (Δ*G*^‡^ in Figure 3A). In methanol, the formation energies of formaldehyde and methyl formate are 17.2 and 10.3 kcal mol^–1^, respectively. This means that overall, the global energy for the formaldehyde decarbonylation from methanol is significantly higher (38.3 kcal mol^–1^) than that from methyl formate (26.8 kcal mol^–1^). The same occurs in toluene, where the formation of formaldehyde and methyl formate is 14.5 and 5.0 kcal mol^–1^, respectively. Therefore, the overall energy barrier for the decarbonylation of formaldehyde from methanol is 32.2 kcal mol^–1,^ and that from methyl formate is 24.4 kcal mol^–1^. These energy values suggest that also through path C, the methanol-to-syngas reaction follows the steps shown in Figure 1, including the formation of methyl formate instead of the direct decarbonylation of formaldehyde. However, formaldehyde can directly decarbonylate if used as the substrate. In addition, the energies found in methanol and toluene suggest that the decarbonylation reaction is slightly favored in toluene, because of the preferred formation of methyl formate in this solvent. This result is consistent with recent experimental work reported using toluene as the main solvent for the methanol decarbonylation reaction.^28^

### Microkinetic Model of the Methanol-to-Syngas Reaction

**Figure 4:**
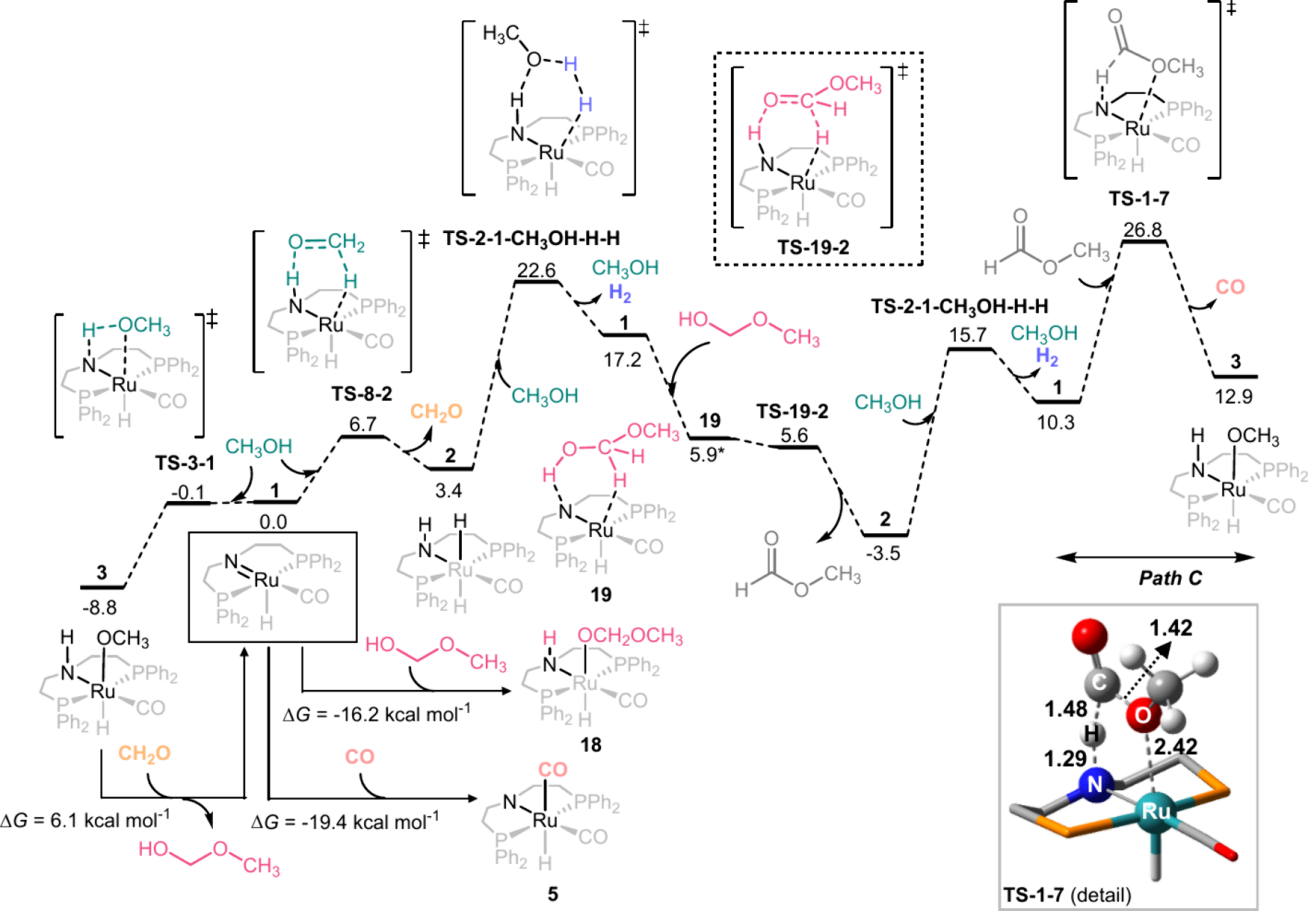
Gibbs Free profile (in kcal mol^–1^) in methanol for the complete methanol-to-syngas reaction catalyzed by **1** via path C. For paths A and B, see Figures S7 and S6. *Optimization in toluene and single-point calculation in methanol.

With the information previously described, we concluded that the formation of syngas from methanol is more likely to proceed via the formation of methyl formate and its decarbonylation through path C. However, the full catalytic cycle is required to determine the key transition states of the reaction, the catalyst resting state, and whether this mechanism agrees with the 2:1 molar ratio observed experimentally. Therefore, the complete mechanism was computed in methanol, toluene, and toluene/methanol for comparison (see the Supporting Information). The complete reaction energy profile in methanol is shown in Figure 4.

The mechanism starts with the reaction of the Ru active catalyst **1** with methanol by two competitive pathways: methanol dehydrogenation yielding formaldehyde, and methanol deprotonation forming the Ru-OCH_3_**3** (Figure 4). This intermediate (**3**) reacts with formaldehyde yielding methoxymethanol, which, like methanol, can react with **1** leading to methyl formate via dehydrogenation or **18** via deprotonation. From these products, only methyl formate can further react with **1** yielding CO via path C. This decarbonylation reaction recovers methoxy intermediate **3**, which needs to dissociate methanol to regenerate active catalyst **1**. The formation of **1** from **2** is also needed after each dehydrogenation process, and it is assisted by methanol. A detailed description of each step is included in the Supporting Information.

**Figure 5:**
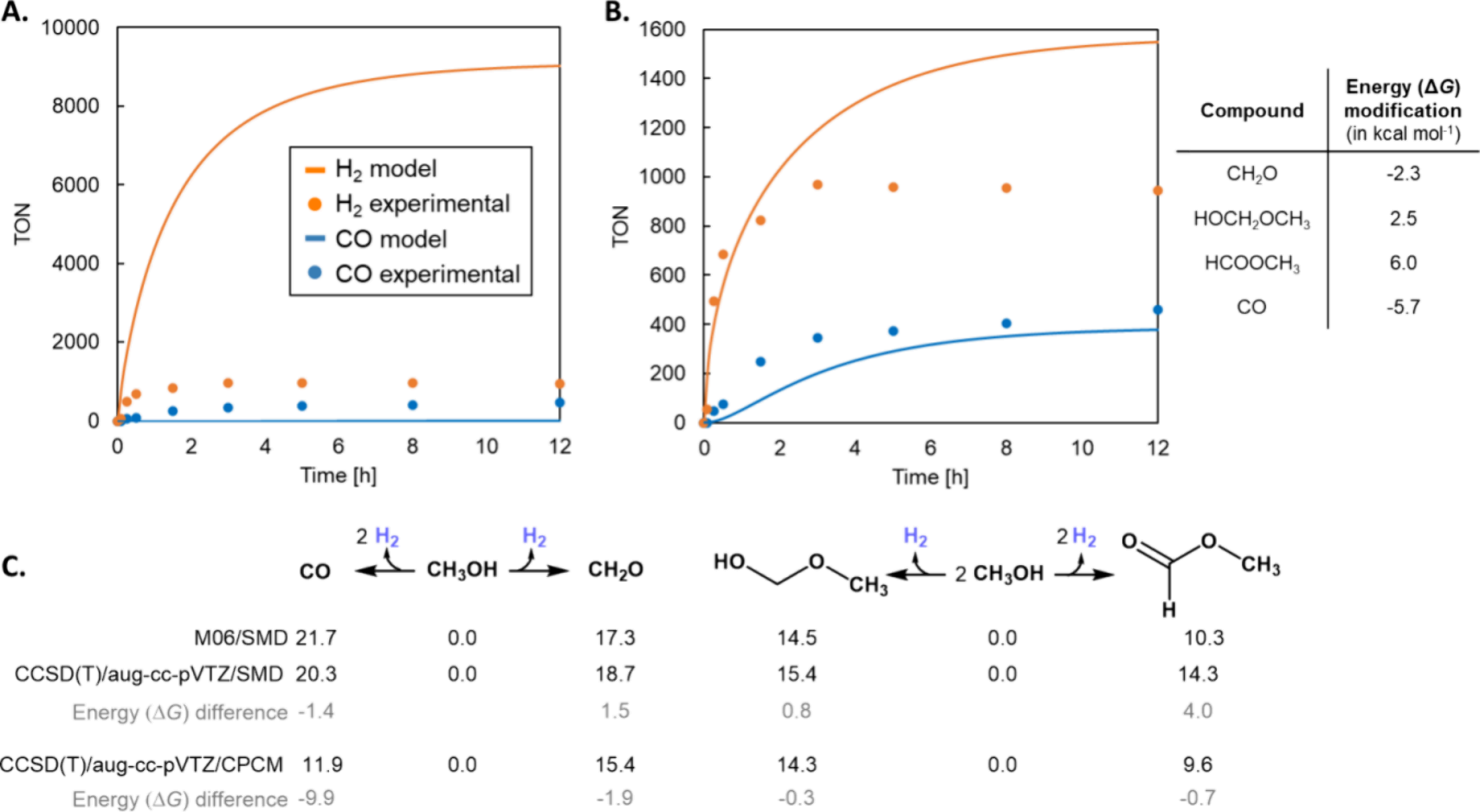
Comparison between the experimental TON of CO and H_2_ produced versus time and DFT computed values for path C (A) without modifications and (B) after introduction of the energy corrections shown in the table to fit the experimental data. (C) Comparison of the thermodynamic values of the organic reactions at M06 and CCSD(T) levels using different solvent models (SMD and CPCM).

The energy profile shows that the highest transition state corresponds to the decarbonylation reaction, favored via path C (see Figures S7 and S6 for the complete paths A and B, respectively). However, it is difficult to predict which intermediates are the most stable and how they affect the production of gaseous products, because of competitive reactions involving the same intermediates. To solve this problem and benchmark our computational results with the experimental data from Leitner et al.’s work,^19^ we built a microkinetic model including all elemental reactions with the computed energies in methanol. This model simulated the batch reactor used by Leitner and co-workers^19^ in the experimental study and included calculations of the concentration of all species in the solution and the gas phase. For this task, we included liquid–vapor equilibrium in the reactor model, assuming the Henry constants for CO and H_2_ in methanol reported by Wu et al.^29^ and using Raoult’s law to estimate the partial pressure of organic compounds in the gas phase (see the Methods section). This assumption is valid due to the huge difference in concentrations between the solvent (methanol) and the solutes. The consideration of the gas phase was critical in this reaction because a considerable amount of methanol would form the vapor phase under the reaction temperature (boiling point of 64.7 °C). Unlike experimental observations, the model predicted a very fast formation of H_2_, while the CO formation was negligible (Figure 5A). A preliminary optimization of all energy values (Ru species, organic molecules, and transition states) and a sensitivity analysis of parameters showed that the free energy of organic molecules influenced the product formation the most (Table S8). The best fitting of the experimental data was obtained by changing the energies of formaldehyde, methoxymethanol, methyl formate, and CO intermediates by −2.3, 2.5, 6.0, and −5.7 kcal mol^–1^, respectively (see Figure 5B). To assign the origin of these energy differences, the results using the M06 method and SMD solvation model were compared with energies obtained using CCSD(T)/aug-cc-pVTZ and CPCM (Figure 5C and Tables S10–S16 for a comparison of computational and experimental data). These results suggest that the solvation model, together with the method in the case of methyl formate, could be responsible for the energy difference. However, applying an energy correction corresponding to the difference between M06/SMD and CCSD(T)/CPCM energies could not reproduce the experimental data (Figure S11). Therefore, we continue our study using the energy fitting obtained with the sensitivity analysis.

**Figure 6:**
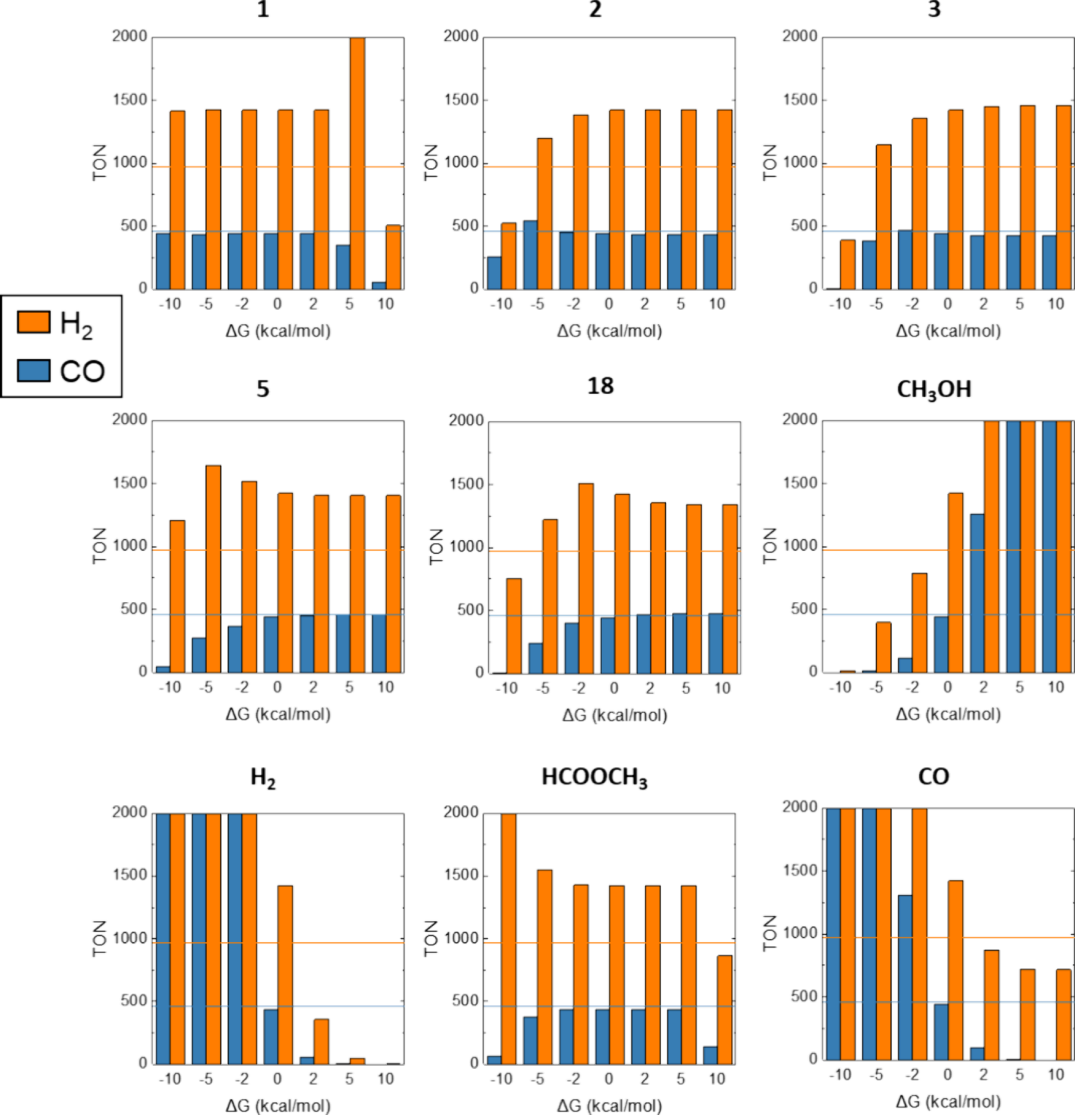
Sensitivity analysis of the free energy of the different intermediates in the TON of H_2_ and CO after reaction for 12 h of reaction. Horizontal lines indicate observed TON values after 12 h by Leitner and co-workers.^19^

**Figure 7:**
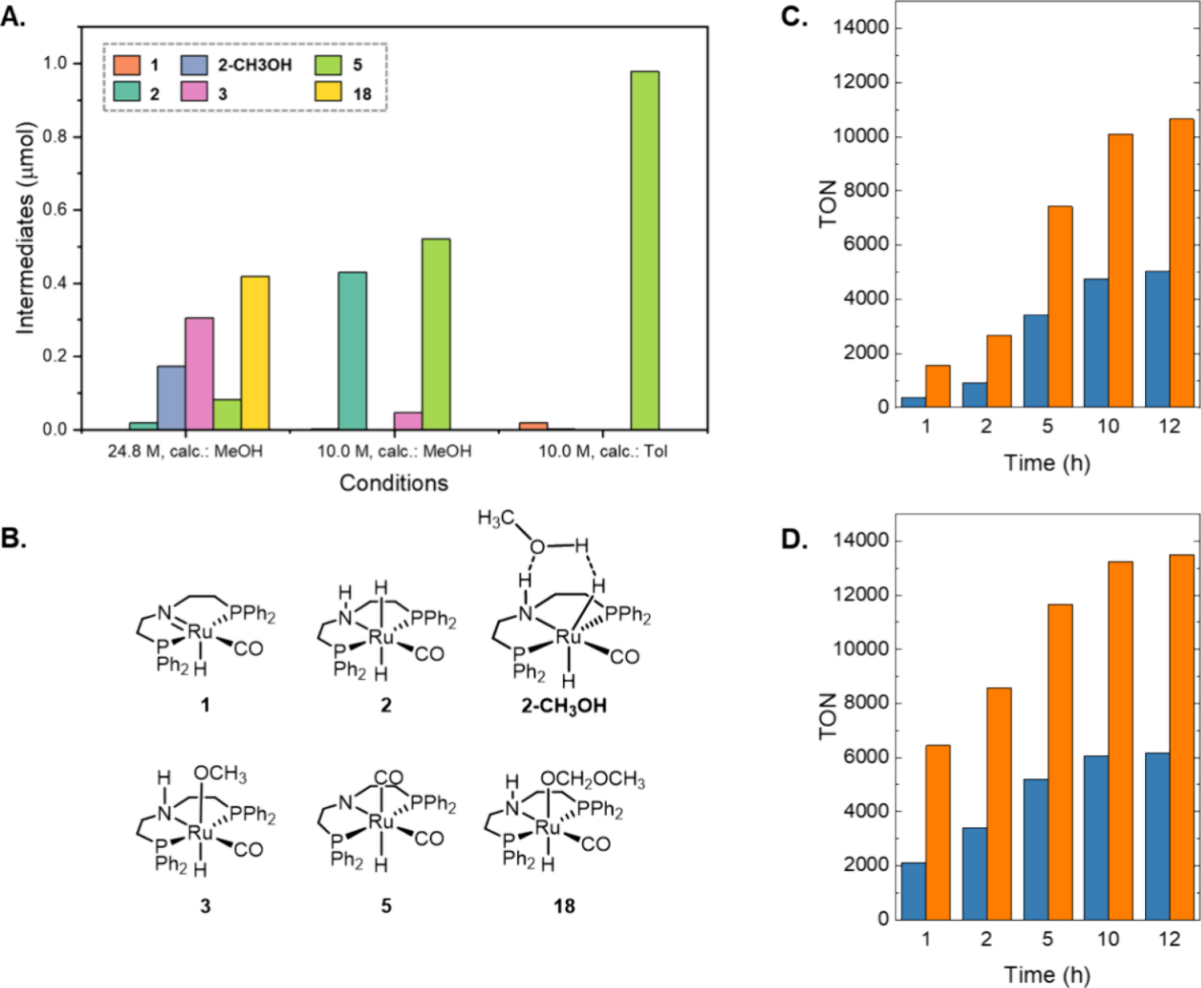
(A) Ru intermediates after 12 h reaction at different conditions. (B) Structure of intermediates. TON of CO and H_2_ over time (C) in methanol and (D) in a 10 M methanol solution in toluene.

After the energy changes for the organic species were included, additional sensitivity analyses of all energy values were performed to evaluate the dependence of the TON and product distribution with all reaction intermediates (Figure 6 and Figure S9). Figure 6 (and Figure S9) shows the comparison of H_2_ and CO TON values after 12 h of reaction. As optimizations suggested before, the most substantial influence was shown by the organic molecules, namely, methanol, CO, and H_2_, which have a huge impact (in the ±2 kcal mol^–1^ range) over gas phase H_2_ and CO production. The large effect indicates that the formation of products is limited by the reaction thermodynamics, which is highly endergonic (Δ*G* = 21.7 kcal mol^–1^, Figure 5C). Formaldehyde has much less influence, as its formation and consumption via reaction with **3** have low energy barriers (Δ*G* ≤ 7 kcal mol^–1^, see Figure 4 and Figure S3). All catalyst intermediates have some influence on the product formation (TON) when an energy shift of −2 kcal mol^–1^. Interestingly, this change may be detrimental for H_2_ and beneficial for CO (as seen for **2** and **3**), or the other way around (as for **5** and **18**). Not surprisingly, all these intermediates have a significant concentration in solution and therefore can be considered as catalyst resting states (see Figure 7A,B). In all cases, bigger changes than −5 kcal mol^–1^ lead to a decrease in CO production. These results suggest that the TON values in CO cannot be increased by modifying the catalyst, because the reaction is limited by the methanol-to-syngas reaction thermodynamics.

A microkinetic model for the methanol-to-syngas reaction was also performed, including path A instead of path C for the decarbonylation of methyl formate. Path B is very similar kinetically to path A, so it was not included in the study. After the corrections for the organic species shown in Figure 5B, the sensitivity analysis over path A show that CO is formed only by destabilizing **4** in ca. 25 kcal mol^–1^ or stabilizing CO in ca. 20 kcal mol^–1^ (most likely a combination of both), which seems unfeasible. This result made us conclude that path C is the preferred pathway, not only because it has a lower energy barrier for the decarbonylation reaction (see Figure 2) but also because it prevents the formation of intermediate **4**, which is highly stable and not observed experimentally.

To analyze the effect of the solvent on the rates of the reactions and the nature of the resting states, we used the microkinetic model for path C with the computed energy values of the species in toluene using the same energy fitting obtained in methanol. In contrast to the results using methanol as solvent, the catalyst resting state using toluene is intermediates **5** (Figure 7A,B) in agreement with experimental results.^19^ In this case, even without an energy correction, CO formation occurs, and **5** is the resting state (see Table S9). This difference is explained by the lower amount of methanol but also by the lower polarity of the media, which changes the stability of reaction intermediates and products. The latter explains the different intermediates observed using 10 M methanol with the energies computed in methanol. In this case, a similar concentration is found for **2** and **5**, with some amount of **3**. The evolution of CO and H_2_ gas formation was also analyzed, and we observed that the reaction was faster in toluene (Figure 7C,D). From this study, we conclude that a higher concentration of methanol could block the catalyst by forming intermediate **3**.

**Figure 8:**
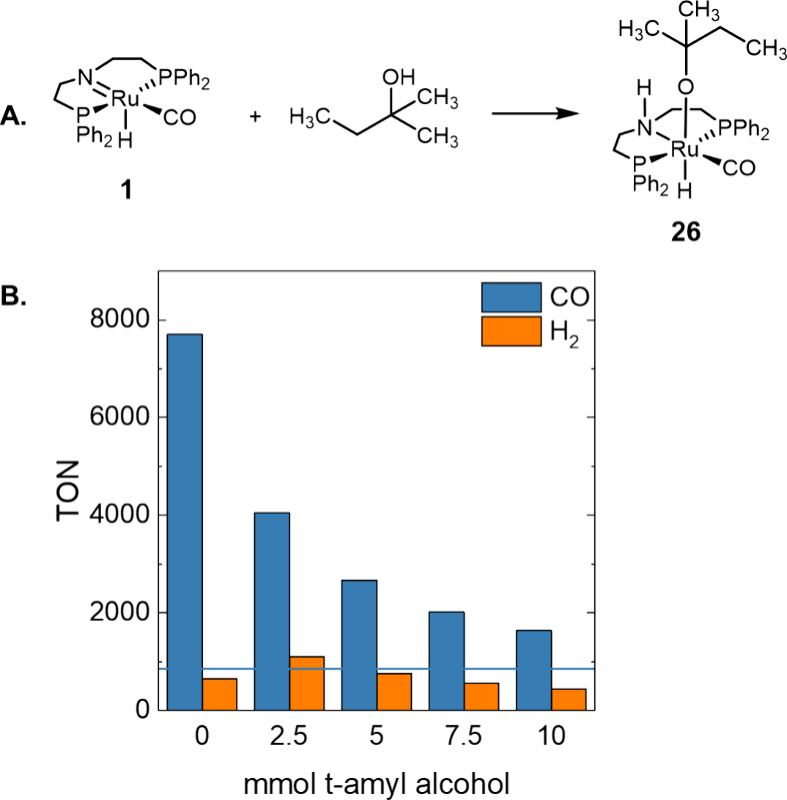
(A) Formation of complex **26** from complex **1** and *t*-amyl alcohol. (B) Analysis of the solvent concentration in the TON of CO and H_2_ after 12 h of reaction using methyl formate as a substrate. The horizontal line indicates the observed TON values after 12 h by Leitner and co-workers using ca. 5 mmol *t*-amyl alcohol.^19^

As methyl formate is proposed as the main source of CO formation, the reaction outcome using methyl formate as a reactant was also investigated. Qualitatively, the results predicted by the model and path C are similar to the experiments, with significantly higher production of CO than H_2_ in this case.^19^ However, the predicted TON values were higher than those observed in the experiments. We hypothesized that the solvent employed (*t*-amyl alcohol) might influence the outcome of the reaction because it could interact with the intermediate **1** in a similar manner to methanol (Figure 8A). To investigate our hypothesis, we computed the formation of complex **26** from **1** and *t*-amyl alcohol, which is thermodynamically favorable by ca. 7.6 kcal mol^–1^. This reaction was incorporated into the microkinetic model, and the inhibition effect of the solvent for the CO production was studied (Figure 8B). Please note that this reaction does not have any impact on the previous results, as reactions were performed in pure methanol. Indeed, CO formation is clearly inhibited by the addition of the solvent, while the production of H_2_ remains almost unchanged. Therefore, our results suggest that methyl formate and the solvent play a key role in the production of CO.

## Conclusions

In this work, the methanol-to-syngas reaction mechanism was studied using DFT calculations and analyzed using microkinetic models. Our results show that the reaction does not go through a Ru–CO_2_CH_3_ intermediate (**4**), as previously proposed.^19^ Instead, the decarbonylation of methyl formate takes place by the direct liberation of CO by a stepwise (in methanol) or concerted (in toluene) reaction, yielding a Ru-OCH_3_ intermediate (**3**). This mechanism is consistent with the fragmentation of methyl formate into H^+^, CO, and CH_3_O^–^ fragments and is preferred over formaldehyde decarbonylation because of the higher stability of methyl formate compared to that of formaldehyde. This reaction is preferred in a nonpolar solvent such as toluene over protic solvents such as methanol or *t*-amyl alcohol because it favors the formation of methyl formate (Figure 3B) and disfavors the formation of alkoxy Ru intermediates, such as **3** or **26**. The simulations of the microkinetic models based on quantum chemical calculations of the reaction mechanism were compared with the experimental data from autoclave experiments using a reactor model that considers the specific reaction setup. This allowed us to further support our hypotheses about the mechanism and shows that the previous considerations are to be discarded in favor of the mechanism presented here.

A sensitivity analysis considering variations in the free energy of all intermediates showed that the H_2_:CO ratio is highly influenced by the energy of the organic reactants, intermediates, and products, which are influenced by the solvent model and computational method. Instead, the energy of the Ru-containing intermediates needs to change by more than 5 kcal mol^–1^ in most cases to impact the TON and product distribution. This information should be relevant for optimizing carbonylation reactions using methanol and other carbonyl-containing groups as CO-surrogate models. In addition, given the similar acid–base properties of Ru–N in **1** with some heterogeneous catalysts, the mechanistic information presented in this work may also be applied to heterogeneous catalysis.

## Methods

### Computational Details

Geometry optimizations were carried out with the M06-L-D3^30,31^ functional, as implemented in the Gaussian16 software package.^32^ Structures were fully optimized without any geometry or symmetry constraints with the double-ζ quality def2-SVP basis set.^33,34^ Vibrational frequencies were computed at the same level of theory to classify all stationary points as either saddle points (transition states, with a single imaginary frequency) or energy minima (reactants, intermediates, and products, with only real frequencies). These calculations were also used to obtain the thermochemistry corrections (zero-point, thermal, and entropy energies) at *P* = 1 atm and *T* = 298.15 K conditions. The energy of the optimized geometries was refined by single-point calculations with the M06-D3^31,35^ functional and triple-ζ quality def2-TZVP basis set for all the Ru complexes and organic species.^33,34^ The M06-D3 functional was selected based on results from a previous study and a benchmark included in the Supporting Information (Table S14).^36^ Solvent effects for methanol or toluene were included in all calculations using the SMD model.^37^ The energies reported in the paper were obtained by adding the thermochemistry corrections to the refined potential energies. In addition, a correction of ±1.9 kcal mol^–1^ was applied to the Gibbs free energy change of reactions involving a change of molecularity for changing the standard state from the gas phase (1 atm) to solution (1 M). Energy refinements for the organic reaction thermodynamics were performed using DLPNO-CCSD(T) calculations withthe cc-PVTZ-DK basis setin ORCA software package (version 6.0.0).^38^

### Microkinetic Modeling

Microkinetic models, including the elementary steps shown in Figure S8 and the vapor–liquid equilibrium transfer, were coupled with an in-house-developed reactor model (implemented in MATLAB). The reaction rate of each elementary step was calculated as
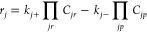
1where *C*_*jr*_ and *C*_*jp*_ are the reactants and products involved in each *j* reaction and *k*_*j+*_ and *k*_*j-*_ are the rate constant of the forward and backward reactions, calculated as
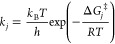
2where *k*_*B*_ is the Boltzmann’s constant, *T* is the reaction temperature, *h* is the Planck’s constant, *R* is the Universal gas constant and Δ*G*_*j*_^‡^ is the free energy barrier of each *j* reaction (forward or backward). Gas phase methanol, methyl formate, CO, and H_2_ were considered in the reactor model for which the gas–liquid equilibrium was assumed using the parameters reported by Wu et al.^29^ Due to the low concentration of CO and H_2_ in the methanol solvent, Henry's law was assumed, thereby computing the partial pressure in the autoclave as
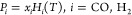
3

Likewise, the partial pressure of methanol (and methyl formate), whose concentration is close to 1 (solvent), was estimated by assuming Raoult’s law of ideal mixtures,
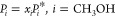
4computing the vapor pressure (*P*_i_*) by using Antoine’s law. The values to apply Antoine’s law were also reported by Wu et al.^29^ The mass transfer resistance between the liquid and the gas phases was assumed to be negligible in all simulations.

To compute the kinetics, the association and dissociation reactions (r01 and r10) were assumed to have low Gibbs energy barriers (Δ*G*^‡^ ≤ 3 kcal mol^–1^), having no effect on the global kinetics of the reaction. The initial concentrations used in the simulation were those reported in the experiments^19^ (i.e., 24.8 M of methanol and 1 mM of catalyst). Simulations were carried out for a total time of 12 h at a temperature of 423.15 K. Table S7 contains the absolute energies of the species involved in the microkinetic model in methanol and toluene.

## Supporting Information Available

Complete energy profiles for the methanol dehydrogenation, catalyst recovery and formation of methyl formate in methanol and toluene, NPA analysis, and details on the microkinetic modeling and energy benchmark (PDF)
Cartesian Coordinates (XYZ)

## Supplementary Material

cs4c06818_si_001.pdf

cs4c06818_si_002.xyz

## References

[ref1] Olah G. A.; Goeppert A.; Prakash G. S. Chemical recycling of carbon dioxide to methanol and dimethyl ether: from greenhouse gas to renewable, environmentally carbon neutral fuels and synthetic hydrocarbons. J. Org. Chem. 2009, 74 (2), 487–498. 10.1021/jo801260f. 19063591

[ref2] Olah G. A.; Goeppert A.; Prakash G. S. Beyond oil and gas: the methanol economy; John Wiley & Sons: 2011.

[ref3] Kumar A.; Daw P.; Milstein D. Homogeneous catalysis for sustainable energy: hydrogen and methanol economies, fuels from biomass, and related topics. Chem. Rev. 2022, 122 (1), 385–441. 10.1021/acs.chemrev.1c00412. 34727501 PMC8759071

[ref4] Sivakumar G.; Kumar R.; Yadav V.; Gupta V.; Balaraman E. Multi-Functionality of Methanol in Sustainable Catalysis: Beyond Methanol Economy. ACS Catal. 2023, 13 (22), 15013–15053. 10.1021/acscatal.3c03957.

[ref5] Sordakis K.; Tang C.; Vogt L. K.; Junge H.; Dyson P. J.; Beller M.; Laurenczy G. Homogeneous catalysis for sustainable hydrogen storage in formic acid and alcohols. Chem. Rev. 2018, 118 (2), 372–433. 10.1021/acs.chemrev.7b00182. 28985048

[ref6] Greeley J.; Mavrikakis M. Methanol decomposition on Cu (111): a DFT study. J. Catal. 2002, 208 (2), 291–300. 10.1006/jcat.2002.3586.

[ref7] Baudais F.; Borschke A.; Fedyk J.; Dignam M. The decomposition of methanol on Ni (100). Surf. Sci. 1980, 100 (1), 210–224. 10.1016/0039-6028(80)90453-7.

[ref8] Kandoi S.; Greeley J.; Sanchez-Castillo M. A.; Evans S. T.; Gokhale A. A.; Dumesic J. A.; Mavrikakis M. Prediction of experimental methanol decomposition rates on platinum from first principles. Top. Catal. 2006, 37, 17–28. 10.1007/s11244-006-0001-1.

[ref9] Greeley J.; Mavrikakis M. A first-principles study of methanol decomposition on Pt (111). J. Am. Chem. Soc. 2002, 124 (24), 7193–7201. 10.1021/ja017818k. 12059245

[ref10] Greeley J.; Mavrikakis M. Competitive paths for methanol decomposition on Pt (111). J. Am. Chem. Soc. 2004, 126 (12), 3910–3919. 10.1021/ja037700z. 15038745

[ref11] Kruse N.; Rebholz M.; Matolin V.; Chuah G.; Block J. H. Methanol decomposition on Pd (111) single crystal surfaces. Surf. Sci. 1990, 238 (1–3), L457–L462. 10.1016/0039-6028(90)90054-C.

[ref12] García-Muelas R.; Li Q.; Lopez N. Density functional theory comparison of methanol decomposition and reverse reactions on metal surfaces. ACS Catal. 2015, 5 (2), 1027–1036. 10.1021/cs501698w.

[ref13] Zhou C.; Ding D.; Zhu W.; Chang X.; Zhang T.; Wu H.; Yang H.; Sun L. Mechanism of formaldehyde advanced interaction and degradation on Fe3O4 (1 1 1) catalyst: Density functional theory study. Appl. Surf. Sci. 2020, 520, 146324 10.1016/j.apsusc.2020.146324.

[ref14] Ma F.-Q.; Lu D.-S.; Guo Z.-Y. Base-catalyzed decomposition of methyl formate to carbon monoxide and methanol over zeolite catalysts. J. Mol. Catal. 1993, 78 (3), 309–325. 10.1016/0304-5102(93)87061-C.

[ref15] Liu Y.; Kirchberger F. M.; Müller S.; Eder M.; Tonigold M.; Sanchez-Sanchez M.; Lercher J. A. Critical role of formaldehyde during methanol conversion to hydrocarbons. Nat. Commun. 2019, 10 (1), 1462 10.1038/s41467-019-09449-7. 30931945 PMC6443648

[ref16] Liu Y.; Müller S.; Berger D.; Jelic J.; Reuter K.; Tonigold M.; Sanchez-Sanchez M.; Lercher J. A. Formation Mechanism of the First Carbon–Carbon Bond and the First Olefin in the Methanol Conversion into Hydrocarbons. Angew. Chem., Int. Ed. 2016, 55 (19), 5723–5726. 10.1002/anie.201511678. 27037603

[ref17] Jing W.; Shen H.; Qin R.; Wu Q.; Liu K.; Zheng N. Surface and interface coordination chemistry learned from model heterogeneous metal nanocatalysts: from atomically dispersed catalysts to atomically precise clusters. Chem. Rev. 2023, 123 (9), 5948–6002. 10.1021/acs.chemrev.2c00569. 36574336

[ref18] Aireddy D. R.; Ding K. Heterolytic Dissociation of H2 in Heterogeneous Catalysis. ACS Catal. 2022, 12 (8), 4707–4723. 10.1021/acscatal.2c00584.

[ref19] Kaithal A.; Chatterjee B.; Werlé C.; Leitner W. Acceptorless dehydrogenation of methanol to carbon monoxide and hydrogen using molecular catalysts. Angew. Chem., Int. Ed. 2021, 60 (51), 26500–26505. 10.1002/anie.202110910. PMC9299216 34596302

[ref20] Geng L.; Zhang M.; Zhang Z.; Li Y. Production of carbon monoxide and hydrogen from methanol using a ruthenium pincer complex: a DFT study. Dalton Trans. 2023, 52 (38), 13653–13661. 10.1039/D3DT01912H. 37702003

[ref21] Yang L.; Guo X.; Ren Y.; Gu R.; Chen Z.-X.; Zeng G. Mechanistic Insight into Acceptorless Dehydrogenation of Methanol to Syngas Catalyzed by MACHO-Type Ruthenium and Manganese Complexes: A DFT Study. Inorg. Chem. 2023, 62 (48), 19516–19526. 10.1021/acs.inorgchem.3c02619. 37966423

[ref22] Nielsen M.; Alberico E.; Baumann W.; Drexler H.-J.; Junge H.; Gladiali S.; Beller M. Low-temperature aqueous-phase methanol dehydrogenation to hydrogen and carbon dioxide. Nature 2013, 495 (7439), 85–89. 10.1038/nature11891. 23446345

[ref23] Alberico E.; Lennox A. J. J.; Vogt L. K.; Jiao H.; Baumann W.; Drexler H.-J.; Nielsen M.; Spannenberg A.; Checinski M. P.; Junge H.; et al. Unravelling the Mechanism of Basic Aqueous Methanol Dehydrogenation Catalyzed by Ru–PNP Pincer Complexes. J. Am. Chem. Soc. 2016, 138 (45), 14890–14904. 10.1021/jacs.6b05692. 27759392

[ref24] Rama R. J.; Nova A.; Nicasio M. C. Microkinetic Model as a Crucial Tool for Understanding Homogeneous Catalysis. ChemCatChem 2024, 16, e202400224 10.1002/cctc.202400224.

[ref25] Besora M.; Maseras F. Microkinetic modeling in homogeneous catalysis. Wiley Interdiscip. Rev.: Comput. Mol. Sci. 2018, 8 (6), e1372 10.1002/wcms.1372.

[ref26] Sciortino G.; Maseras F. Microkinetic modelling in computational homogeneous catalysis and beyond. Theor. Chem. Acc. 2023, 142 (10), 99 10.1007/s00214-023-03044-2.

[ref27] Konishi H.; Matsubara M.; Mori K.; Tokiwa T.; Arulmozhiraja S.; Yamamoto Y.; Ishikawa Y.; Hashimoto H.; Shigeta Y.; Tokiwa H.; et al. Mechanistic Insight into Weak Base-Catalyzed Generation of Carbon Monoxide from Phenyl Formate and Its Application to Catalytic Carbonylation at Room Temperature without Use of External Carbon Monoxide Gas. Adv. Synth. Catal. 2017, 359 (20), 3592–3601. 10.1002/adsc.201700751.

[ref28] Bonde A.; Jakobsen J. B.; Ahrens A.; Huang W.; Jackstell R.; Beller M.; Skrydstrup T. Integrating Hydroformylations with Methanol-to-Syngas Reforming. Chem 2025, 102396 10.2139/ssrn.4960740.

[ref29] Wu F.; Zhao Q.; Tao L.; Danaci D.; Xiao P.; Hasan F. A.; Webley P. A. Solubility of carbon monoxide and hydrogen in methanol and methyl formate: 298–373 K and 0.3–3.3 MPa. J. Chem. Eng. Data 2019, 64 (12), 5609–5621. 10.1021/acs.jced.9b00676.

[ref30] Zhao Y.; Truhlar D. G. A new local density functional for main-group thermochemistry, transition metal bonding, thermochemical kinetics, and noncovalent interactions. J. Chem. Phys. 2006, 125 (19), 194101 10.1063/1.2370993. 17129083

[ref31] Grimme S.; Antony J.; Ehrlich S.; Krieg H. A consistent and accurate ab initio parametrization of density functional dispersion correction (DFT-D) for the 94 elements H-Pu. J. Chem. Phys. 2010, 132 (15), 154104 10.1063/1.3382344. 20423165

[ref32] Frisch M. J.; Trucks G. W.; Schlegel H. B.; Scuseria G. E.; Robb M. A.; Cheeseman J. R.; Scalmani G.; Barone V.; Petersson G. A.; Nakatsuji H.; Li X.; Caricato M.; Marenich A. V.; Bloino J.; Janesko B. G.; Gomperts R.; Mennucci B.; Hratchian H. P.; Ortiz J. V.; Izmaylov A. F.; Sonnenberg J. L.; Williams; Ding F.; Lipparini F.; Egidi F.; Goings J.; Peng B.; Petrone A.; Henderson T.; Ranasinghe D.; Zakrzewski V. G.; Gao J.; Rega N.; Zheng G.; Liang W.; Hada M.; Ehara M.; Toyota K.; Fukuda R.; Hasegawa J.; Ishida M.; Nakajima T.; Honda Y.; Kitao O.; Nakai H.; Vreven T.; Throssell K.; Montgomery J. A. Jr.; Peralta J. E.; Ogliaro F.; Bearpark M. J.; Heyd J. J.; Brothers E. N.; Kudin K. N.; Staroverov V. N.; Keith T. A.; Kobayashi R.; Normand J.; Raghavachari K.; Rendell A. P.; Burant J. C.; Iyengar S. S.; Tomasi J.; Cossi M.; Millam J. M.; Klene M.; Adamo C.; Cammi R.; Ochterski J. W.; Martin R. L.; Morokuma K.; Farkas O.; Foresman J. B.; Fox D. J. Gaussian 16 *Rev. B.01*, Gaussian, Inc.: Wallingford, CT, 2016.

[ref33] Weigend F. Accurate Coulomb-fitting basis sets for H to Rn. Phys. Chem. Chem. Phys. 2006, 8 (9), 1057–1065. 10.1039/b515623h. 16633586

[ref34] Weigend F.; Ahlrichs R. Balanced basis sets of split valence, triple zeta valence and quadruple zeta valence quality for H to Rn: Design and assessment of accuracy. Phys. Chem. Chem. Phys. 2005, 7 (18), 3297–3305. 10.1039/b508541a. 16240044

[ref35] Zhao Y.; Truhlar D. G. The M06 suite of density functionals for main group thermochemistry, thermochemical kinetics, noncovalent interactions, excited states, and transition elements: two new functionals and systematic testing of four M06-class functionals and 12 other functionals. Theor. Chem. Acc. 2008, 120 (1), 215–241. 10.1007/s00214-007-0310-x.

[ref36] Artús Suàrez L.; Culakova Z.; Balcells D.; Bernskoetter W. H.; Eisenstein O.; Goldberg K. I.; Hazari N.; Tilset M.; Nova A. The Key Role of the Hemiaminal Intermediate in the Iron-Catalyzed Deaminative Hydrogenation of Amides. ACS Catal. 2018, 8 (9), 8751–8762. 10.1021/acscatal.8b02184.

[ref37] Marenich A. V.; Cramer C. J.; Truhlar D. G. Universal solvation model based on solute electron density and on a continuum model of the solvent defined by the bulk dielectric constant and atomic surface tensions. J. Phys. Chem. B 2009, 113 (18), 6378–6396. 10.1021/jp810292n. 19366259

[ref38] Neese F.; Wennmohs F.; Becker U.; Riplinger C. The ORCA quantum chemistry program package. J. Chem. Phys. 2022, 152 (22), 224108 10.1063/5.0004608. 32534543

