# Supplementary material for: Outer-Sphere CO Release Mechanism in the Methanol-to-Syngas Reaction Catalyzed by a Ru-PNP Pincer Complex
Source: ACS Catal. 2025 Mar 12;15(6):5113–22. doi: 10.1021/acscatal.4c06818 (PMC11934088; doi:10.1021/acscatal.4c06818)
Supplement: Supplementary file 1 — cs4c06818_si_001.pdf [file cs4c06818_si_001.pdf]

Supporting Information

Outer-sphere CO release mechanism in the methanol to syngas reaction catalyzed by a Ru-PNP pincer complex

Jiali Liu,[a,b] Raquel J. Rama,[c,*] Tomás Cordero-Lanzac,[c], Mohamed E. A. Safy,[d] Robert Franke,[a,b] and Ainara Nova[c,d,*]

[a] *Evonik Oxeno GmbH & Co. KG, Paul-Baumann-Str. 1, 45772 Marl, Germany*
[b] *Lehrstuhl für Theoretische Chemie, Ruhr-Universität Bochum, 44780 Bochum, Germany*
[c] *Center for Materials Science and Nanotechnology (SMN), Department of Chemistry, University of Oslo, 0315 Oslo, Norway*
[d] *Hylleraas Centre for Quantum Molecular Sciences, Department of Chemistry, University of Oslo, N-0315 Oslo*

*Correspondence to r.j.rama@smn.uio.no and a.n.flores@kjemi.uio.no

Table of Contents

Methanol dehydrogenation to formaldehyde....S2

Catalyst recovery or dehydrogenation....S3

Formation of methyl formate....S4

NPA analysis....S7

Microkinetic modeling....S8

Energy benchmark....S12

References....S15

Methanol dehydrogenation to formaldehyde

The dehydrogenation of methanol by complex **1** was studied using methanol as a solvent. The TSs that could not be located in methanol were estimated by performing the geometry optimization in toluene and a single-point calculation in methanol. The formation of formaldehyde by reaction of **1** with methanol is endergonic by 17.2 kcal mol^{-1} (Figure S1). The release of formaldehyde goes via a stepwise outer-sphere mechanism, with the highest energy barrier being 6.7 kcal mol^{-1} (**TS-8-2**, Pathway I). A competitive reaction is the formation of ruthenium methoxide (**3**) through a concerted proton transfer (Pathway II). This reaction is barrierless and thermodynamically favorable by 8.8 kcal mol^{-1}, consistent with the reported detection of ruthenium alkoxides in the presence of alcohol.[1, 2]

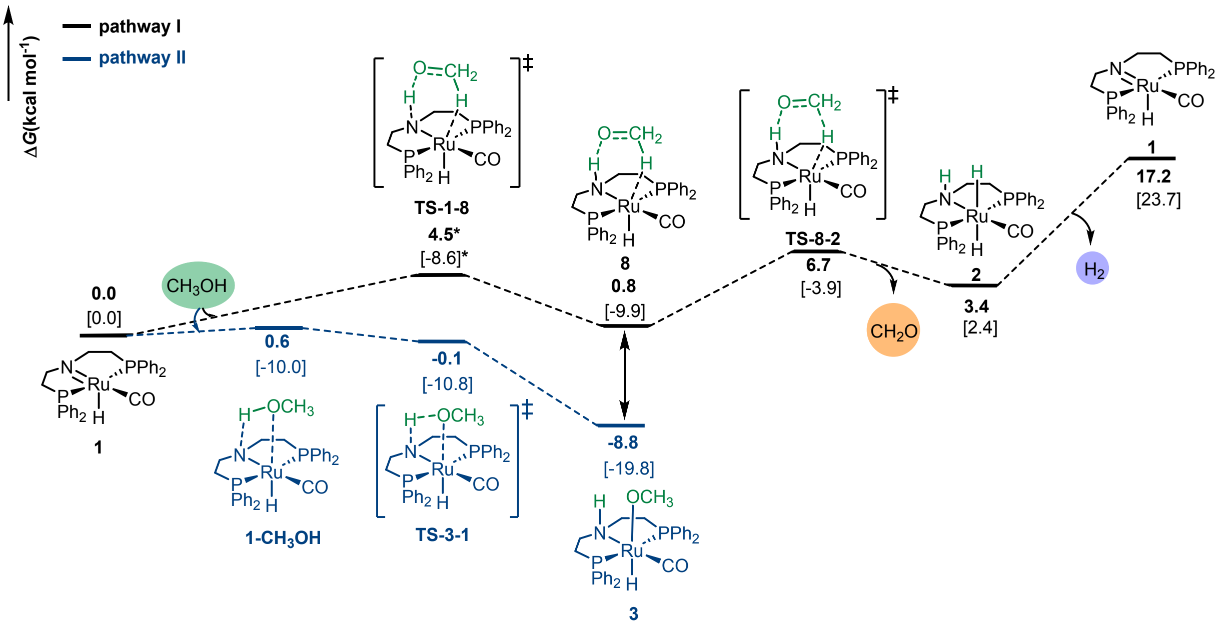

Figure S1. Gibbs energy profile for the dehydrogenation of methanol to formaldehyde (methanol-opt/methanol-SP). Values in [] represent reaction enthalpy; * denotes optimization in toluene and single-point calculation in methanol.

Table S1. Gibbs free energy values for the dehydrogenation of methanol to formaldehyde: toluene-optimized structures with single-point energy corrections in methanol and toluene (kcal mol^{-1}). Values in [] represent reaction enthalpy.

Species	toluene-opt/methanol-SP	toluene-opt/toluene-SP
**1**	0.0 [0.0]	0.0 [0.0]
**1-CH_3OH**	2.5 [-9.6]	0.0 [-12.0]
**TS-1-8**	4.5 [-8.6]	3.4 [-9.8]
**8**	4.9 [-8.2]	5.5 [-7.7]
**TS-3-1**	3.8 [-8.4]	0.6 [-11.6]
**3**	-5.8 [-17.9]	-5.3 [-17.4]
**TS-8-2**	9.4 [-3.0]	7.3 [-5.1]

**2**	6.0 [3.3]	7.7 [4.9]
**1**	17.2 [23.7]	14.5 [21.0]

A comparison of the computed free energies in methanol and toluene shows that the formation of formaldehyde is slightly preferred in toluene over methanol, with energy differences ranging from 0.5 to 4.3 kcal mol^{-1} (see Table S1). In addition, some intermediates give significant differences when using methanol or toluene for geometry optimizations, followed by single-point calculations in methanol (*e. g.* 4.1 kcal mol^{-1} in **8**). This result indicates that methanol has an impact on the geometries for these particular transformations.

Catalyst recovery or dehydrogenation

The concerted dehydrogenation of complex **2** to yield **1** has an energy barrier of 28.2 kcal mol^{-1} (see Figure S2). The methanol-assisted dehydrogenation of complex **2** was also evaluated. This reaction takes place through the protonation of the hydride by methanol (**TS-2-1-CH_3OH-H-H**), leading to a Ru-dihydrogen intermediate (**2-CH_3OH-H_2**) followed by the deprotonation of the amine ligand (**TS-2-1-CH_3OH-H^+**). From these two processes, the formation of **2-CH_3OH-H_2** has the highest energy barrier, which is 19.2 kcal mol^{-1}.

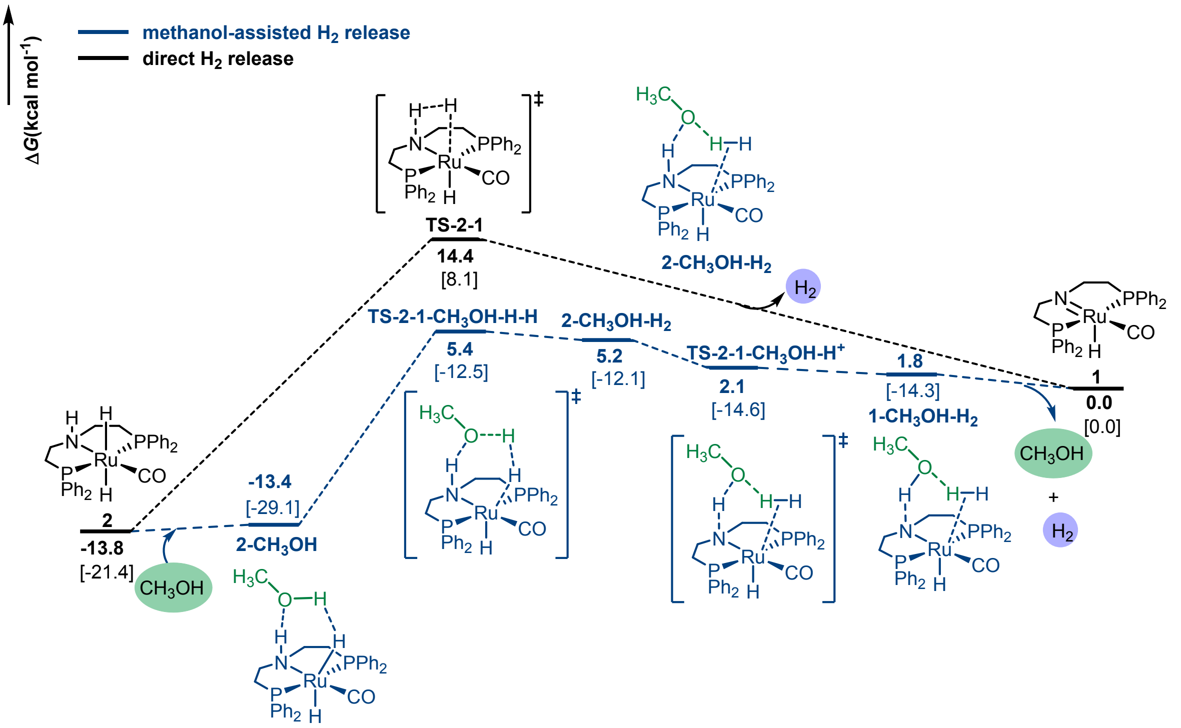

Figure S2. Gibbs energy profile for the catalyst activation (methanol-opt/methanol-SP). Values in [] represent reaction enthalpy.

In this case, the solvent has also a significant impact on the reaction mechanism, and instead of a stepwise process, the methanol-assisted pathway is concerted in toluene. The interaction of methanol with complex **2** gave intermediate **2-CH_3OH** (-3.9 kcal mol^{-1}), which released a H_2 molecule rendering complex **1** and regenerating methanol with an energy barrier of 18.0 kcal mol^{-1} via a concerted transition state **TS-2-1-CH_3OH** (shown in Table S2). With the single-point correction in methanol, the effective Gibbs free energy barrier is 19.7 kcal mol^{-1}.
Overall, the lower energy barrier found for the methanol-assisted route suggested that methanol could facilitate the catalyst activation, which is consistent with previous studies.[3, 4]

Table S2. Gibbs free energy values for the catalyst activation: toluene-optimized structures with single-point energy corrections in methanol and toluene (kcal mol^{-1}). Values in [] represent reaction enthalpy.

Species	toluene-opt/methanol-SP	toluene-opt/toluene-SP
**2**	-11.1 [-20.4]	-6.9 [-16.2]
**TS-2-1**	16.6 [8.6]	15.4 [7.4]
**1**	0.0 [0.0]	0.0 [0.0]
**2-CH$_3$OH**	-10.6 [-28.0]	-10.8 [-28.2]
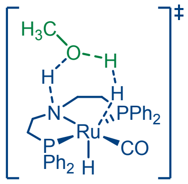 **TS-2-1-CH$_3$OH**	8.6 [-9.9]	7.2 [-11.2]
**1-CH$_3$OH-H$_2$**	3.4 [-13.4]	0.7 [-16.1]

Formation of methyl formate

The formation of methoxymethanol, by the coupling of formaldehyde and methanol, has been proposed in prior studies.[5, 6] A concerted mechanism has been located in toluene solvent, where species **3** reacts with formaldehyde to form methoxymethanol. However, this concerted transition state, **TS-3-1-CH$_2$O**, could not be obtained in methanol solvent. The values marked with an asterisk (*) in Figure S3 represent the transition state optimized in toluene, with single-point electronic energy corrections performed in methanol. This is a barrierless process via **TS-3-1-CH$_2$O**. In toluene (Table S3), the generation of methoxymethanol is also barrierless. Thermodynamically, this reaction is preferred in toluene rather than methanol solvent (3.1 kcal mol^{-1} vs. 6.1 kcal mol^{-1}).

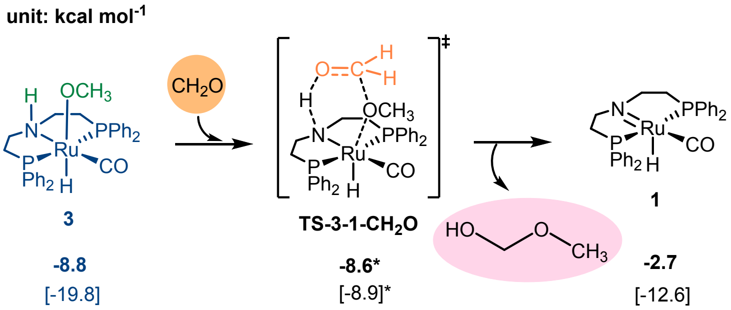

Figure S3. Ru-catalyzed formation of methoxymethanol (methanol-opt/methanol-SP). Values in [] represent reaction enthalpy; * denotes optimization in toluene and single-point calculation in methanol.

Table S3. Gibbs free energy values for the Ru-catalyzed formation of methoxymethanol: toluene-optimized structures with single-point energy corrections in methanol and toluene (kcal mol^{-1}). Values in [] represent reaction enthalpy.

Species	toluene-opt/methanol-SP	toluene-opt/toluene-SP
**3**	-5.8 [-17.9]	-5.3 [-17.4]

**TS-3-1-CH$_2$O**	-7.7 [-8.9]	-10.7 [-14.6]
**1**	-2.4 [-12.3]	-2.2 [-12.1]

As displayed in Figure S4 and Table S4, the subsequent dehydrogenation of the alcohol by **1** was also found to be barrierless, yielding methyl formate, which was detected by NMR during the reaction progress.[7]

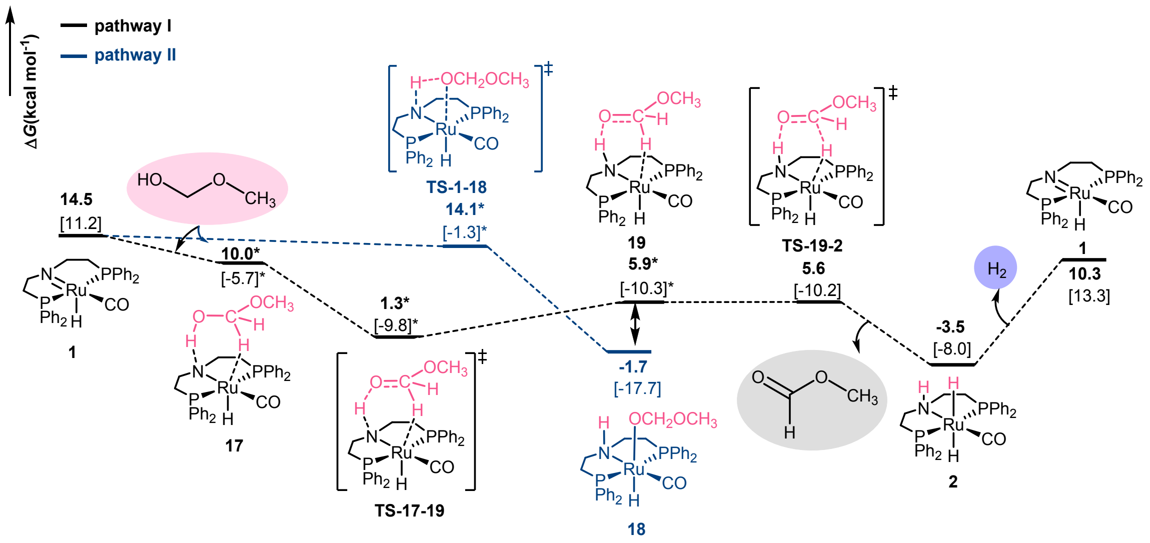

Figure S4. Gibbs energy profile for the dehydrogenation of methoxymethanol to methyl formate (methanol-opt/methanol-SP). Values in [] represent reaction enthalpy; * denotes optimization in toluene and single-point calculation in methanol.

Table S4. Gibbs free energy values for the dehydrogenation of methoxymethanol to methyl formate: toluene-optimized structures with single-point energy corrections in methanol and toluene (kcal mol^{-1}). Values in [] represent reaction enthalpy.

Species	toluene-opt/methanol-SP	toluene-opt/toluene-SP
**1**	17.2 [11.4]	14.5 [8.9]
**17**	10.9 [-5.7]	7.0 [-9.7]
**TS-17-19**	7.2 [-9.8]	9.1 [-7.9]
**19**	6.7 [-10.3]	6.1 [-11.0]
**TS-1-18**	15.0 [-1.3]	9.8 [-6.4]
**18**	1.6 [-13.8]	0.1 [-15.4]
**TS-19-2**	8.6 [-9.0]	5.2 [-12.5]
**2**	-0.8 [-7.0]	-1.9 [-8.0]
**1**	10.3 [13.4]	5.0 [8.1]

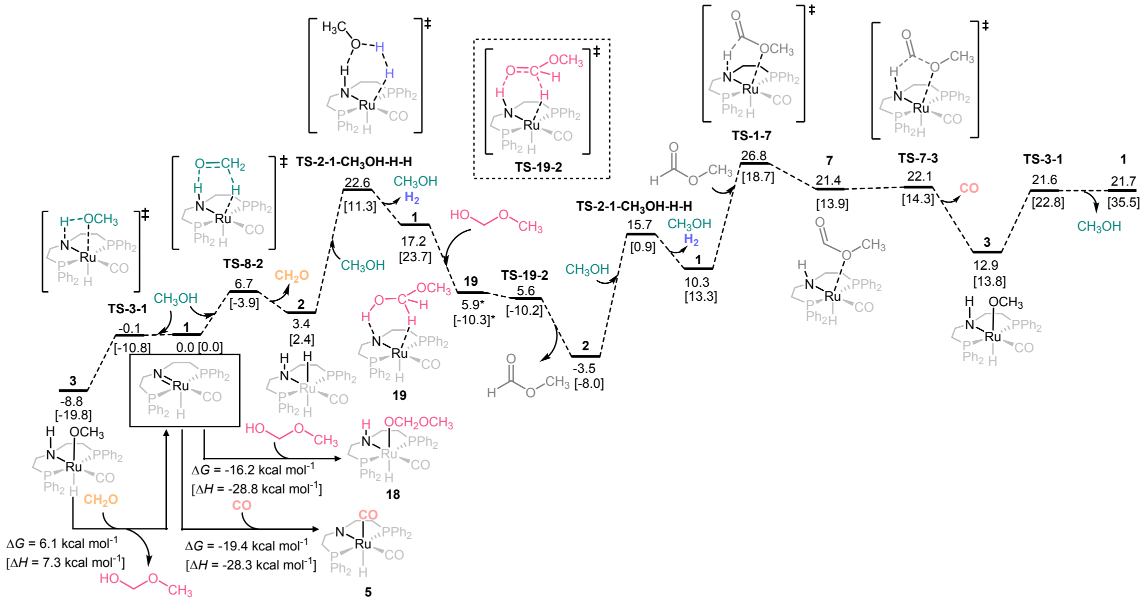

Figure S5. Gibbs Free profile (in kcal mol^{-1}) in methanol for the methanol to syngas reaction catalyzed by **1** via **Path C**. Values in [] represent reaction enthalpy; * denotes optimization in toluene and single-point calculation in methanol.

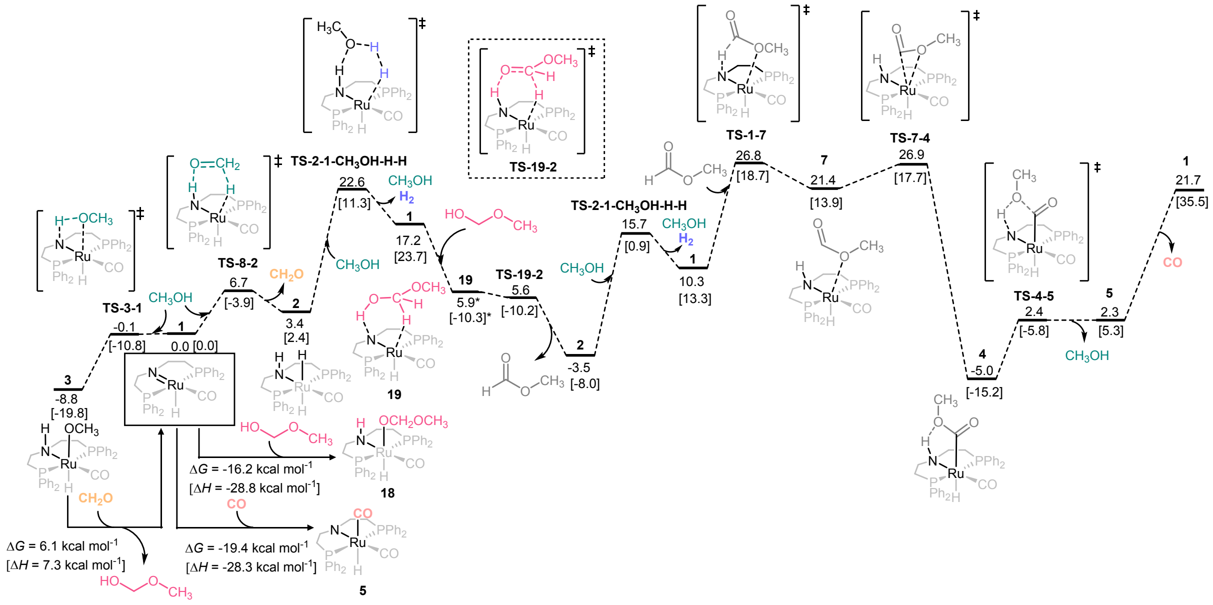

Figure S6. Gibbs Free profile (in kcal mol^{-1}) in methanol for the methanol to syngas reaction catalyzed by **1** via **Path B**. Values in [] represent reaction enthalpy; * denotes optimization in toluene and single-point calculation in methanol.

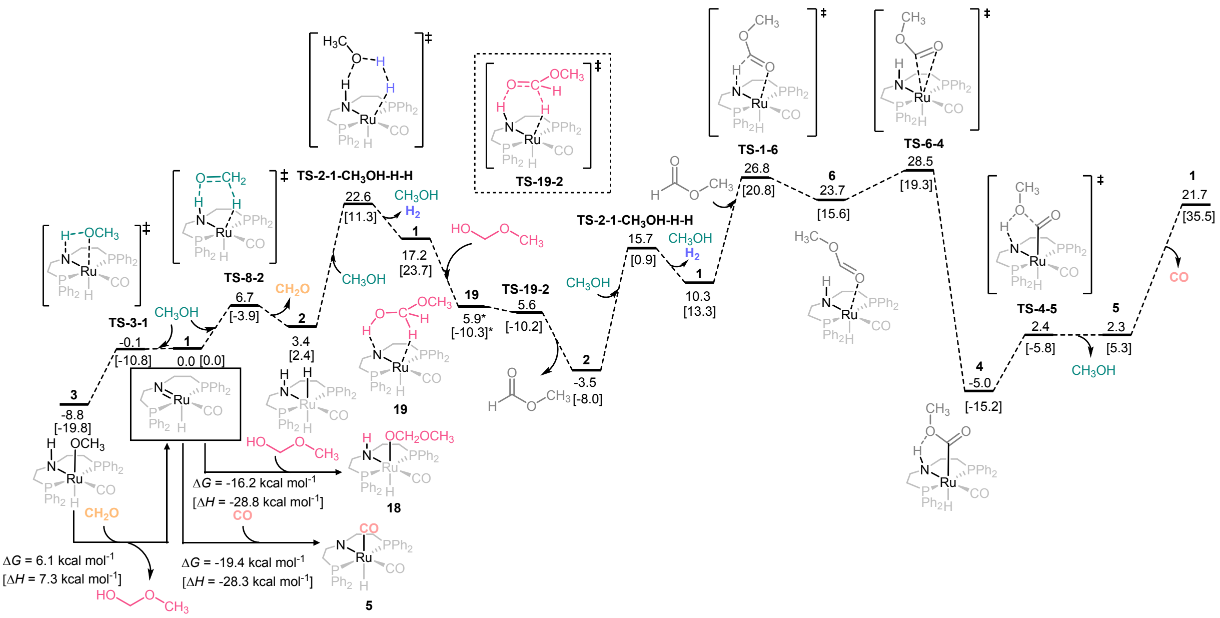

Figure S7. Gibbs Free profile (in kcal mol-1) in methanol for the methanol to syngas reaction catalyzed by 1 via **Path A**. Values in [] represent reaction enthalpy; * denotes optimization in toluene and single-point calculation in methanol.

NPA analysis

Table S5. NPA analysis of key atoms in structures optimized in toluene solvent.

	**1**	**CH_2O**	**TS-1-2-CO**	*Δq*
N	-0.642		-0.716	-0.074
Ru	-1.026		-1.160	-0.134
H^1		0.107	0.294	0.187
C		0.307	0.255	-0.052
O		-0.521	-0.512	0.009
H^2		0.107	0.051	-0.056
sum(N, Ru)				-0.208
sum(C, O)				-0.043

	**1**	**$HCOOCH_3$**	**TS-1-3-CO**	*Δq*
N	-0.642		-0.751	-0.109
Ru	-1.026		-0.908	0.118
H^1		0.123	0.325	0.202
C^1		0.708	0.500	-0.208
O^1		-0.615	-0.635	-0.02
O^2		-0.567	-0.610	-0.043
C^2		-0.249	-0.245	0.004
H^2, H^3, H^4 (CH_3 of OCH_3)		0.198 0.198 0.205	0.198 0.192 0.185	-0.026
sum(N, Ru)				0.009
sum(C^1, O^1)				-0.228
sum(OCH_3)				-0.065

Table S6. NPA analysis of key atoms in structures optimized in methanol solvent.

	**1**	**CH_2O**	**TS-1-2-CO**	*Δq*
N	-0.648		-0.695	-0.047
Ru	-0.961		-1.108	-0.147
H^1		0.122	0.297	0.175

C		0.333	0.231	-0.102
O		-0.577	-0.569	0.008
H^2		0.122	0.072	-0.05
sum(N, Ru)				-0.194
sum(C, O)				-0.094

	**1**	**$HCOOCH_3$**	**TS-1-7**	*Δq*
N	-0.648		-0.753	-0.105
Ru	-0.961		-0.862	0.099
H^1		0.142	0.319	0.177
C^1		0.730	0.527	-0.203
O^1		-0.669	-0.710	-0.041
O^2		-0.568	-0.590	-0.022
C^2		-0.254	-0.255	-0.001
H^2, H^3, H^4 (CH_3 of OCH_3)		0.203 0.203 0.213	0.194 0.200 0.204	-0.021
sum(N, Ru)				-0.006
sum(C^1, O^1)				-0.244
sum(OCH_3)				-0.044

Microkinetic modeling

Table S7 contains the absolute energies of the species involved in the microkinetic model, which includes the reactions presented in Figure S8. TSs of reactions r06 and r07 were not found in methanol as solvent, so the barrier of reactions r06 and r07 were based on the values optimized in toluene with single-point calculation in methanol.

Paths A and B	Path C
r01: **2** + $CH_3OH \rightleftharpoons$ **2-CH_3OH**	r01: **2** + $CH_3OH \rightleftharpoons$ **2-CH_3OH**
r02: **2-CH_3OH** $\rightleftharpoons$ **1** + H_2 + CH_3OH	r02: **2-CH_3OH** $\rightleftharpoons$ **1** + H_2 + CH_3OH
r03: **1** + $CH_3OH \rightleftharpoons$ **3**	r03: **1** + $CH_3OH \rightleftharpoons$ **3**
r04: **1** + $CH_3OH \rightleftharpoons$ **2** + CH_2O	r04: **1** + $CH_3OH \rightleftharpoons$ **2** + CH_2O
r05: **2** $\rightleftharpoons$ **1** + H_2	r05: **2** $\rightleftharpoons$ **1** + H_2
r06: **3** + $CH_2O \rightleftharpoons$ **1** + $HOCH_2OCH_3$	r06: **3** + $CH_2O \rightleftharpoons$ **1** + $HOCH_2OCH_3$
r07: **1** + $HOCH_2OCH_3 \rightleftharpoons$ **18**	r07: **1** + $HOCH_2OCH_3 \rightleftharpoons$ **18**
r08: **1** + $HOCH_2OCH_3 \rightleftharpoons$ **2** + $HCOOCH_3$	r08: **1** + $HOCH_2OCH_3 \rightleftharpoons$ **2** + $HCOOCH_3$
r09: **1** + $HCOOCH_3 \rightleftharpoons$ **4**	r09: **1** + $HCOOCH_3 \rightleftharpoons$ **3** + CO
r10: **1** + CO $\rightleftharpoons$ **5**	r10: **1** + CO $\rightleftharpoons$ **5**
r11: **1** + $CH_2O \rightleftharpoons$ **2** + CO	r11: **1** + $CH_2O \rightleftharpoons$ **2** + CO
r12: **4** $\rightleftharpoons$ **5** + CH_3OH	

Figure S8. Reactions used in the microkinetic model.

Table S7. Computed absolute energies for species involved in microkinetic modeling.

	$G_{corr.}$/kcal mol^{-1}	
Species	Methanol	Toluene
**1**	-1273403.826	-1273405.447
**2**	-1274151.118	-1274145.659
**2-CH$_3$OH**	-1346736.882	-1346732.686
**3**	-1345998.899	-1345993.91
**4**	-1417114.406	-1417109.48
**5**	-1344520.883	-1344524.463
**18**	-1417844.594	-1417838.336
**CH$_3$OH**	-72588.14862	-72585.03012
**H$_2$**	-735.3493792	-735.2171453
**CO**	-71099.53646	-71102.51504
**CH$_2$O**	-71837.45258	-71837.16389
**HOCH$_2$OCH$_3$**	-144426.4188	-144422.4961
**HCOOCH$_3$**	-143697.2047	-143696.5136
***t*-amyl alcohol**	-171166.0055	-171162.831
**26**	-1444575.532	-1444571.113
**TS-2-1-CH3OH-H-H** (r02)	-1346718.143	-1346714.673
**TS-3-1** (r03)	-1345990.188	-1345987.975
**TS-8-2** (r04)	-1345983.349	-1345981.261
**TS-2-1** (r05)	-1274122.838	-1274123.38
**TS-19-2** (r08)	-1417837.32	-1417833.247
**TS-6-4** (r09, Path A)	-1417080.942	-1417073.52
**TS-1-7** (r09, Path B)	-1417082.575	-
**TS-1-3-CO** (r09, Path C)	-1417082.61	-1417080.673
**TS-1-2-CO** (r11)	-1345218.237	-1345223.055

To evaluate the contribution to the overall production rate, the formation of CO from formaldehyde (r11) was considered in all mechanisms (**Paths A**, **B** and **C**). Comparing the reaction rates of r09 (of **Path C**), r10 and r11, this reaction pathway is more unlikely.

Analysis of Path C: We performed a sensitivity analysis of all energy values in methanol following **Path C** (Table S8). As shown in Table S8, only the energy modification of organic species (methanol, CO and H_2) generates CO gas. It is important to note that the variation of the stability of Ru intermediates does not influence the formation of CO.

Table S8. Sensitivity analysis of the free energy of different species in the TON of H_2 and CO after 12 h of reaction. The color code indicates the accuracy of the model to predict experimental data.

	**H$_2$ TON values after 12 h**							**CO TON values after 12 h**						
	Energy modification (kcal mol^{-1})							Energy modification (kcal mol^{-1})						
Species	**-10**	**-5**	**-2**	**0**	**2**	**5**	**10**	**-10**	**-5**	**-2**	**0**	**2**	**5**	**10**
**1**	8895	9086	9089	9087	9081	11534	1357	0	0	0	0	0	3	0
**2**	1472	8470	8727	9087	9306	9340	9211	0	0	0	0	0	0	0
**2-CH$_3$OH**	193	8321	9245	9087	9042	9037	9037	0	0	0	0	0	0	0
**3**	633	8659	9204	9087	9011	9000	8969	0	0	0	0	0	0	0
**5**	9223	9258	9138	9087	9079	9078	9078	0	0	0	0	0	0	0
**18**	2871	12172	9444	9087	9045	9041	9041	0	3	0	0	0	0	0
**CH$_2$O**	8598	9085	9087	9087	9087	9087	8930	0	0	0	0	0	0	0
**CH$_3$OH**	20	403	1950	9087	13319	13378	13062	0	0	0	0	90	94	28
**CO**	14237	9171	9089	9087	9087	8358	9026	2300	53	1	0	0	0	0
**H$_2$**	12680	12680	12721	9087	1888	180	4	101	101	96	0	0	0	0
**HCOOCH$_3$**	12529	12535	12876	9087	4171	1481	725	0	0	3	0	0	0	2
**HOCH$_2$OCH$_3$**	5786	8996	9085	9087	9082	8651	1340	0	0	0	0	0	0	0

We fitted free energy values of organic intermediates using experimental data. Using the data containing the energy modifications of the fitting (see table in Figure 5B), we performed a sensitivity analysis of the free energy of the different organic compounds and Ru-containing species (Figures 6 and S9). As a result, the effect of the organic molecules was found to be higher than that of the Ru-containing species.

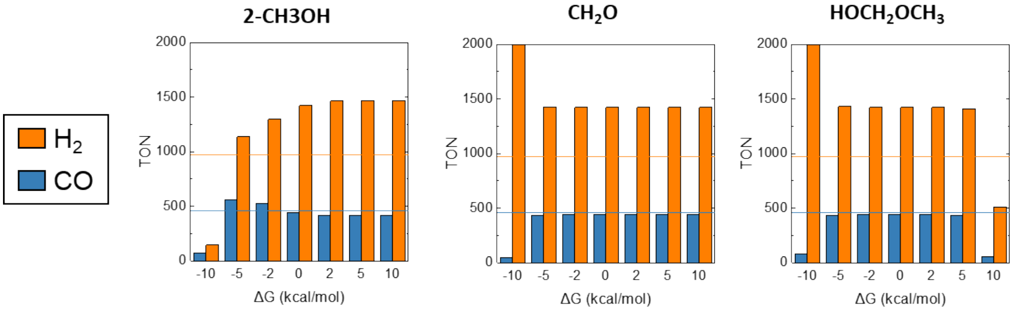

Figure S9. Sensitivity analysis of the free energy of the different species in the TON of H_2 and CO after 12 h of reaction using corrected energy values. Horizontal lines indicate observed TON values after 12 h by Leitner et al.[7].

The reaction following **Path C** was also studied using toluene as a solvent. In contrast to the reaction in methanol, CO was formed without applying any correction, although in a very low concentration compared to H_2 (TON 28). A sensitivity analysis reveals the destabilization of intermediate **5** considerably increases the CO formation, indicating that **5** is the catalyst resting state (Table S9).

Table S9. Sensitivity analysis of the free energy of the different intermediates in the TON of H_2 and CO after 12 h of reaction in toluene. The color code indicates the accuracy of the model experimental data in methanol for comparison with previous tables.

	**H_2 TON values after 12 h**							**CO TON values after 12 h**						
	Energy modification (kcal mol^{-1})							Energy modification (kcal mol^{-1})						
Species	**-10**	**-5**	**-2**	**0**	**2**	**5**	**10**	**-10**	**-5**	**-2**	**0**	**2**	**5**	**10**
**1**	3817	6979	7682	7683	7558	7288	4995	1	5	7	28	31	30	19
**2**	3348	7341	7714	7683	7674	7668	5815	0	2	25	28	29	29	22
**2-CH_3OH**	471	7716	7695	7683	7663	7646	7645	0	26	28	28	28	29	29
**3**	1911	7587	7711	7683	7674	7672	7097	0	2	27	28	28	29	29
**5**	7584	7591	7622	7683	7807	8255	11142	0	1	8	28	97	566	3736
**18**	7720	7686	7701	7683	7683	7683	7683	24	28	28	28	28	28	28
**CH_2O**	7748	7682	7698	7683	7683	7681	5811	31	28	33	28	28	28	22
**CH_3OH**	270	1754	5637	7683	7798	7805	7804	0	4	24	28	29	28	28
**CO**	11534	8262	7791	7683	7633	7630	7684	4126	574	96	28	8	1	0
**H_2**	7428	7426	7512	7683	7562	6403	40	30	30	30	28	27	6	0
**$HCOOCH_3$**	7412	7410	7721	7683	6760	5068	4036	0	0	1	28	94	427	1418
**$HOCH_2OCH_3$**	7683	7684	7684	7683	7678	7568	5518	28	28	28	28	28	28	20

Analysis of Path A: The model using **Path A** predicted a very fast formation of H_2 and no formation of CO with the initially computed data (Figure S10A). After the corrections made by fitting the energy values of organic molecules using experimental data (see Figure 5B), the H_2 production was closer to the experimental values, but no CO formation was observed (Figure S10B). Sensitivity analysis reveals that CO is only formed through this pathway by

destabilizing compound **4** by ca. 25 kcal mol^{-1} or stabilizing CO in ca. 20 kcal mol^{-1} (most likely a combination of both), which seems unfeasible.

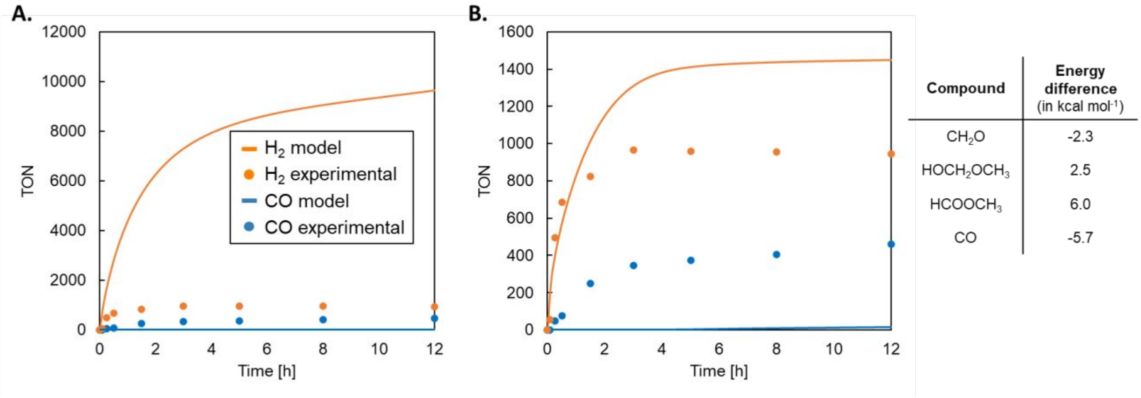

Compound	Energy difference (in kcal mol^{-1})
CH_2O	-2.3
$HOCH_2OCH_3$	2.5
$HCOOCH_3$	6.0
CO	-5.7

Figure S10. Comparison between experimental TON of CO and H_2 produced versus time and modeled values of the **Path A** (A) without modifications and (B) after modifications shown in the table.

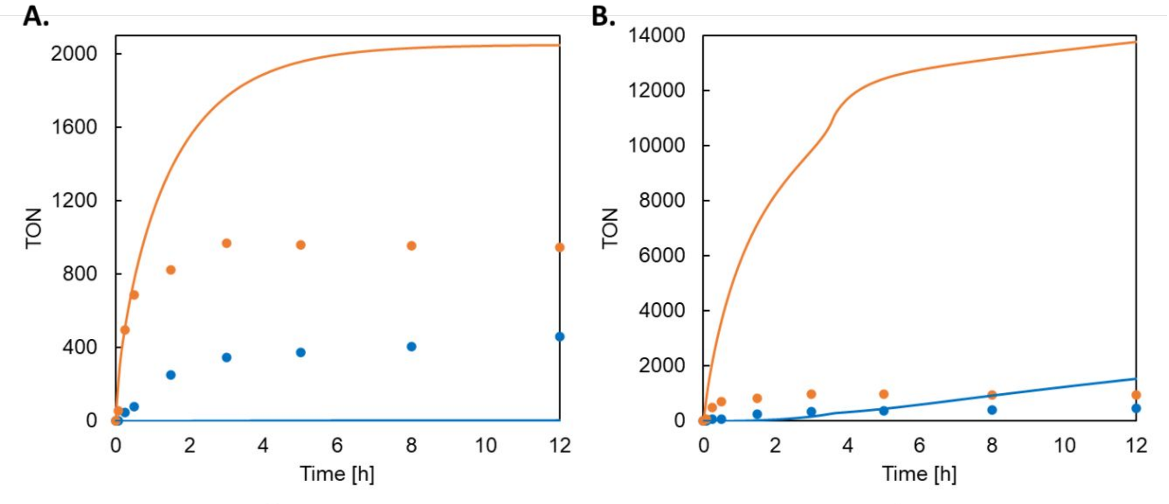

Compound	Energy (ΔG) modification (in kcal mol^{-1})
CH_2O	1.5
$HOCH_2OCH_3$	0.8
$HCOOCH_3$	4.0
CO	-1.4

Compound	Energy (ΔG) modification (in kcal mol^{-1})
CH_2O	-1.9
$HOCH_2OCH_3$	-0.3
$HCOOCH_3$	-0.7
CO	-9.9

Figure S11. Comparison between experimental TON of CO and H_2 produced versus time and modeled values of the **Path C** with energy fitting corresponding to CCSD(T)/aug-cc-pVTZ/SMD (A) and CCSD(T)/aug-cc-pVTZ/CPCM (B). The energy modifications are shown in the tables under the graphs (see Energy benchmark section below for further details).

Analysis of intermediates: The evolution of the reaction intermediates in methanol and toluene was studied using microkinetic models. Simulations were carried out for a total time of 12 hours at a temperature of 423.15 K in a 14 mL autoclave using 1 mL total volume and 1 mM of catalyst. To analyze the influence of the polarity and concentration of solvent, we studied

simulations in pure methanol (24.8 M) or diluted methanol (10 M) in toluene. The simulations in diluted methanol (10 M) were performed using the computed values in methanol (Figure S12B) and toluene (Figure S12C) using the same corrections as in the study in methanol (see Figures S10B and Figure 5B for corrections).

Different resting states were observed using pure methanol (Figure S12A) and diluted methanol (Figure S12B). In toluene, intermediate **5** was the Ru-containing species with higher concentration during the reaction. These results indicate that not only the methanol concentration but also the polarity of the solvent influences the reaction outcome.

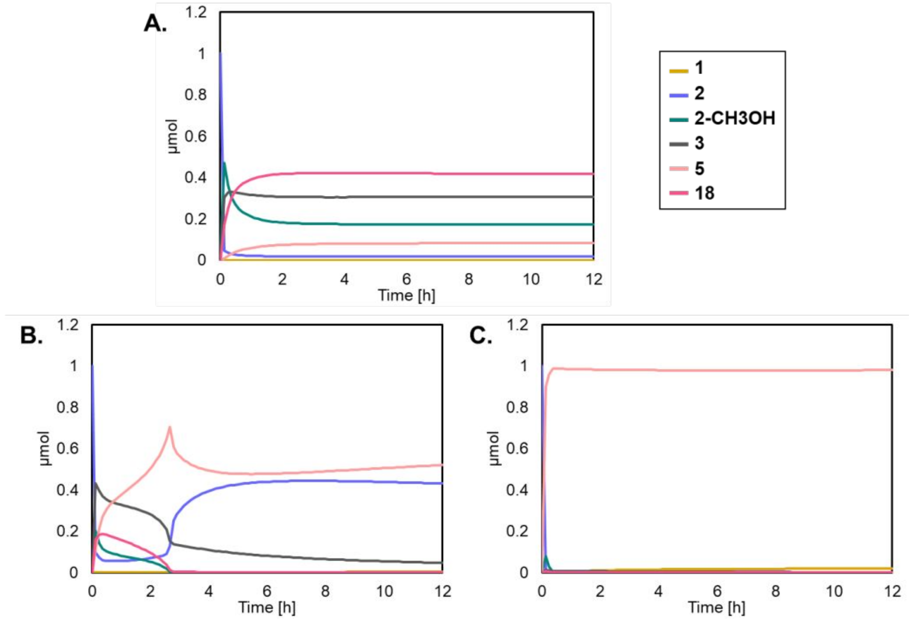

Figure S12. Evolution of the Ru-containing species over time, at 24.8 M of methanol using energies computed in methanol (A); at 10 M of methanol in toluene using energies computed in methanol (B); at 10 M of methanol in toluene using energies computed in toluene (C), all of them including the energy modifications shown in the table of Figure S10B.

Energy benchmark

The sensitivity analysis indicates that the relative energies between methanol, CO and H_2 highly determine the amount of CO generation. Therefore, we decided to analyze how much the methanol to syngas thermodynamics change when using different computational methods and solvation models.

Firstly, the cc-pVTZ[8, 9] and def2-QZVPP[10, 11] basis sets were tested in comparison to the def2-TZVP[10, 11] basis set. As presented in Table S10, changes in the basis set using single-point calculation had a small influence on the methanol to syngas reaction Gibbs free energy in both methanol and toluene solvents.

Table S10. Gibbs free energy for the methanol to syngas reaction in methanol and toluene using M06-D3/BS2//M06L-D3/def2-SVP (kcal mol^{-1}).

BS2	def2-TZVP	cc-pVTZ	def2-QZVPP

methanol-opt/methanol-SP	21.7	22.1	23.0
toluene-opt/toluene-SP	15.9	16.5	17.4

The next step was to test the method and the basis set also for geometry optimization. For this, we selected hybrid (M06[12], M06-2X[12], B3LYP[13] and PBE1PBE[14, 15]) in addition to the non-hybrid functional (M06-L[16]) with GD3[17] dispersion correction using methanol as solvent. As shown in Table S11, there was no difference when changing the basis set from cc-pVTZ to def2-QZVPP for all tested functionals. Similar thermodynamics were obtained employing M06, M06-2X and B3LYP functionals, which were in a range of 21.4 to 23.1 kcal mol^{-1}, close to the result of 21.7 kcal mol^{-1} (Table S10) obtained with the methodology used for the complete catalytic cycle (see methods section in the manuscript). A more endergonic reaction, by about 25 kcal mol^{-1} and 31 kcal mol^{-1}, was obtained using M06-L and PBE1PBE functionals, respectively.

Table S11. Gibbs free energy for the methanol to syngas reaction in methanol using different methods and basis set (BS1) (kcal mol^{-1}).

Method/BS1	M06-D3	M06-L-D3	M06-2X-D3	B3LYP-D3	PBE1PBE-D3
cc-pVTZ	22.1	25.3	22.4	21.4	30.9
def2-QZVPP	23.0	25.1	23.1	22.5	31.7

Since DFT methods did not seem to favor the methanol to syngas reaction as suggested by the sensitivity analysis performed with the MKM, we also tested coupled-cluster (CC) methods and, in particular, the CCSD(T) approach. Geometries and thermodynamic corrections were derived from DFT calculation, while electronic energies were refined using single-point calculations at the CCSD(T)/cc-pVTZ level. In addition, to evaluate the accuracy of both M06 and CCSD(T) energies, the computed thermodynamic data for the methanol to syngas reaction was compared with experimental values for the $CH_3OH \rightarrow CO + 2H_2$ reaction[18] (Table S12). With DFT, the reaction enthalpy is 22.7 kcal mol^{-1}, which is 1 kcal mol^{-1} higher than the experimental value of 21.7 kcal mol^{-1}, while with CCSD(T), using the largest aug-cc-pVTZ basis set, it is 21.2 kcal mol^{-1}, 0.5 kcal mol^{-1} lower than the experimental data. Therefore, there is not a large difference between the two methods.

Table S12. Reaction enthalpy (ΔH) and Gibbs free energy change (ΔG) for the methanol to syngas reaction in the gas phase using M06 and CCSD(T) electronic corrections compared to experimental data (kcal mol^{-1}).

	Exp. data	M06	DLPNO-CCSD(T)	DLPNO-CCSD(T)	CCSD(T)				
Basis set		def2-TZVP	cc-pVTZ	aug-pVTZ	cc-pVTZ	cc-pVQZ	cc-pV5Z	cc-pV6Z	aug-cc-pVTZ
ΔH	21.7	22.7	18.6	21.3	18.6	20.3	21.1	21.2	21.2
ΔG	6.1	7.0	2.8	5.6	2.8	4.6	5.3	5.4	5.4

A similar comparison was done with methyl formate (Table S13), which was also proposed to differ significantly (by 6.0 kcal mol^{-1}) from the computed data, based on the sensitivity analysis shown in Table S8.

Table S13. Reaction enthalpy (ΔH) for the methanol to methylformate and H_2 reaction in the gas phase using DFT and DLPNO-CCSD(T)/aug-pVTZ compared to experimental data (kcal mol^{-1}).

	Exp. data	M06	DLPNO-CCSD(T)
Basis set		def2-TZVP	aug-pVTZ
ΔH	10.1	6.1	10.9

With this information, we used DLPNO-CCSD(T)/aug-cc-pVTZ-DK calculations to benchmark the M06 functional for the two highest energy barriers of the catalytic reaction. The results are presented in Table S14.

Table S14. Gibbs free energy changes in methanol: electronic energy corrections by different methods (kcal mol^{-1}).

ΔG	M06-D3/ def2-TZVP	M06-L-D3/ def2-TZVP	DLPNO-CCSD(T)/ cc-pVTZ-DK	DLPNO-CCSD(T)/ aug-cc-pVTZ-DK
**1+2CH_3OH → TS-2-1-CH_3OH-H-H + CH_2O**	22.6	22.4	22.2	20.0
**1+2CH_3OH → TS-1-7 + 2H_2**	26.8	31.3	27.5	26.3
**CH_3OH → CO + 2H_2**	21.7	25.6	17.7	20.3

We were also interested in the influence of the solvent model on the final energies. Tables S15 and S16 show the energies using SMD, PCM, and CPCM.

Table S15. Gibbs free energy changes for the organic intermediates in the methanol to syngas reaction in methanol using M06/def2-TZVP electronic energy corrections (kcal mol^{-1}) applying SMD, IEFPCM and CPCM solvation model, and the difference between them.

Solvation Model	2CH_3OH	CH_3OH+CH_2O+H_2	$HOCH_2OCH_3$+H_2	$HCOOCH_3$+2H_2	CH_3OH+2H_2+CO
**SMD**	0.0	17.3	14.5	10.3	21.7
**IEFPCM**	0.0	13.8	13.1	5.4	13.3
**CPCM**	0.0	13.8	13.1	5.4	13.3
**$\Delta G_{IEFPCM-SMD}$**	0.0	-3.5	-1.4	-4.9	-8.4
**$\Delta G_{CPCM-SMD}$**	0.0	-3.4	-1.4	-4.9	-8.4

Table S16. Gibbs free energy changes for the organic intermediates in the methanol to syngas reaction in methanol using CCSD(T)/aug-cc-pVTZ electronic energy corrections (kcal mol^{-1}) applying SMD, IEFPCM and CPCM solvation model, and the difference between them.

Solvation Model	$2CH_3OH$	$CH_3OH+CH_2O+H_2$	$HOCH_2OCH_3+H_2$	$HCOOCH_3+2H_2$	CH_3OH+2H_2+CO
SMD	0.0	18.7	15.4	14.3	20.3
IEFPCM	0.0	15.4	14.3	9.5	11.8
CPCM	0.0	15.4	14.3	9.6	11.9
$\Delta G_{\text{IEFPCM-SMD}}$	0.0	-3.4	-1.1	-4.8	-8.5
$\Delta G_{\text{CPCM-SMD}}$	0.0	-3.4	-1.1	-4.7	-8.4

References

(1) Kaithal, A.; Schmitz, M.; Hölscher, M.; Leitner, W. On the Mechanism of the Ruthenium-catalyzed β-methylation of Alcohols with Methanol. *ChemCatChem* **2020**, *12* (3), 781-787.

(2) Alberico, E.; Lennox, A. J.; Vogt, L. K.; Jiao, H.; Baumann, W.; Drexler, H.-J.; Nielsen, M.; Spannenberg, A.; Checinski, M. P.; Junge, H. Unravelling the mechanism of basic aqueous methanol dehydrogenation catalyzed by Ru–PNP pincer complexes. *J. Am. Chem. Soc.* **2016**, *138* (45), 14890-14904.

(3) Yang, X. Mechanistic insights into ruthenium-catalyzed production of H2 and CO2 from methanol and water: a DFT study. *ACS Catal.* **2014**, *4* (4), 1129-1133.

(4) Kuß, D. A.; Hölscher, M.; Leitner, W. Combined Computational and Experimental Investigation on the Mechanism of CO2 Hydrogenation to Methanol with Mn-PNP-Pincer Catalysts. *ACS Catal.* **2022**, *12* (24), 15310-15322.

(5) Geng, L.; Zhang, M.; Zhang, Z.; Li, Y. Production of carbon monoxide and hydrogen from methanol using a ruthenium pincer complex: a DFT study. *Dalton Trans.* **2023**, *52* (38), 13653-13661.

(6) Yang, L.; Guo, X.; Ren, Y.; Gu, R.; Chen, Z.-X.; Zeng, G. Mechanistic Insight into Acceptorless Dehydrogenation of Methanol to Syngas Catalyzed by MACHO-Type Ruthenium and Manganese Complexes: A DFT Study. *Inorg. Chem.* **2023**, *62* (48), 19516-19526.

(7) Kaithal, A.; Chatterjee, B.; Werlé, C.; Leitner, W. Acceptorless dehydrogenation of methanol to carbon monoxide and hydrogen using molecular catalysts. *Angew. Chem. Int. Ed.* **2021**, *60* (51), 26500-26505.

(8) Kendall, R. A.; Dunning, T. H., Jr.; Harrison, R. J. Electron affinities of the first-row atoms revisited. Systematic basis sets and wave functions. *J. Chem. Phys.* **1992**, *96* (9), 6796-6806.

(9) Dunning, T. H., Jr. Gaussian basis sets for use in correlated molecular calculations. I. The atoms boron through neon and hydrogen. *J. Chem. Phys.* **1989**, *90* (2), 1007-1023.

(10) Weigend, F. Accurate Coulomb-fitting basis sets for H to Rn. *Phys. Chem. Chem. Phys.* **2006**, *8* (9), 1057-1065.

(11) Weigend, F.; Ahlrichs, R. Balanced basis sets of split valence, triple zeta valence and quadruple zeta valence quality for H to Rn: Design and assessment of accuracy. *Phys. Chem. Chem. Phys.* **2005**, *7* (18), 3297-3305.

(12) Zhao, Y.; Truhlar, D. G. The M06 suite of density functionals for main group thermochemistry, thermochemical kinetics, noncovalent interactions, excited states, and transition elements: two new functionals and systematic testing of four M06-class functionals and 12 other functionals. *Theor. Chem. Acc.* **2008**, *120* (1), 215-241.

(13) Becke, A. D. Density-functional thermochemistry. III. The role of exact exchange. *J. Chem. Phys.* **1993**, *98* (7), 5648-5652.

(14) Adamo, C.; Barone, V. Toward reliable density functional methods without adjustable parameters: The PBE0 model. *J. Chem. Phys.* **1999**, *110* (13), 6158-6170.

(15) Ernzerhof, M.; Scuseria, G. E. Assessment of the Perdew–Burke–Ernzerhof exchange-correlation functional. *J. Chem. Phys.* **1999**, *110* (11), 5029-5036.

(16) Zhao, Y.; Truhlar, D. G. A new local density functional for main-group thermochemistry, transition metal bonding, thermochemical kinetics, and noncovalent interactions. *J. Chem. Phys.* **2006**, *125* (19), 194101.

(17) Grimme, S.; Antony, J.; Ehrlich, S.; Krieg, H. A consistent and accurate ab initio parametrization of density functional dispersion correction (DFT-D) for the 94 elements H-Pu. *J. Chem. Phys.* **2010**, *132* (15), 154104.
(18) Linstorm, P. NIST chemistry webbook, NIST standard reference database number 69. *J. Phys. Chem. Ref. Data, Monograph* **1998**, *9*, 1-1951.
